# Copper-catalyzed [3 + 2] cycloaddition of (phenylethynyl)di-*p*-tolylstibane with organic azides

**DOI:** 10.3762/bjoc.12.123

**Published:** 2016-06-23

**Authors:** Mizuki Yamada, Mio Matsumura, Yuki Uchida, Masatoshi Kawahata, Yuki Murata, Naoki Kakusawa, Kentaro Yamaguchi, Shuji Yasuike

**Affiliations:** 1 School of Pharmaceutical Sciences, Aichi Gakuin University, 1-100 Kusumoto-cho, Chikusa-ku, Nagoya 464-8650, Japan; 2 Pharmaceutical Sciences at Kagawa Campus, Tokushima Bunri University, 1314-1 Shido, Sanuki, Kagawa 769-2193, Japan; 3 Faculty of Pharmaceutical Sciences, Hokuriku University, Ho-3 Kanagawa-machi, Kanazawa 920-1181, Japan

**Keywords:** cycloaddition, copper catalyst, ethynylstibane, organic azide, 1,2,3-triazole

## Abstract

Trisubstituted 5-stibano-1*H*-1,2,3-triazoles were synthesized in moderate to excellent yields by the Cu-catalyzed [3 + 2] cycloaddition of a ethynylstibane with organic azides in the presence of CuBr (5 mol %) under aerobic conditions. The reaction of 5-stibanotriazole with HCl, I_2_, and NOBF_4_ afforded 1-benzyl-4-phenyltriazole, 1-benzyl-5-iodo-4-phenyltriazole, and a pentavalent organoantimony compound, respectively.

## Introduction

The 1,3-dipolar azide–alkyne cycloaddition (AAC) has been effective for the synthesis of a wide variety of 1,2,3-triazoles [1]. However, this reaction has some limitations such as the requirement of high temperature and the generation of regioisomers. In 2002 Sharpless [2] and Meldal [3] independently reported that the addition of catalytic amounts of copper reagent in the AAC allow the reaction to proceed under milder reaction conditions, and there was also an effect on the regioselectivity for the synthesis of 1,4-disubstituted 1,2,3-triazoles. Since then, the CuAAC has been widely applied in organic synthesis [4–12], molecular biology [13–17], and materials science [18–20]. There are many reports of CuAACs by using terminal alkynes (including metal acetylides) [4–20]. But the use of internal alkynes in CuAACs for the synthesis of 1,4,5-trisubstituted 1,2,3-triazoles is a more challenging area because of the difficulty in regiocontrol based on the increased steric hindrance [21–22]. A regioselective CuAAC synthesis of fully substituted 1,2,3-triazoles having group 15 (P, Bi) elements as substituents at the C-5 position was recently attempted. Li et al. reported that the cycloaddition of alkynylphosphonate with benzyl azide did not generate 1,2,3-triazolyl-5-phosphonates, but a three-component reaction of a terminal alkyne, an organic azide, and an H-phosphate in the presence of CuCl_2_ (10 mol %) and triethylamine (2 equiv) afforded the desired 1,2,3-triazolyl-5-phosphonates [23]. Fokin et al. carried out the reaction of ethynylbismuthane with organic azides using CuOTf (5 mol %) and isolated 5-bismuthano-1,2,3-triazoles (Bi), which could be employed as versatile building blocks in chemical synthesis [24]. One drawback of the Cu-catalyzed cycloaddition of alkynylbismuthanes is the requirement of alkyne derivatives based on the phenothiabismuthane 5,5-dioxide framework for stabilization. The utility of organoantimony compounds in organic synthesis has attracted much interest during the last two decades [25–26]. Trivalent organoantimony compounds (stibanes) such as aryl- and ethynylstibanes are useful transmetalation agents in Pd-catalyzed cross-coupling reactions with aryl halides and acyl chlorides [27–32]. Stibanes have many advantages such as the handle ability without special care, low toxicity, and availability. Therefore, the synthesis and reactivity of novel stibanes are important for the development of effective organic reagents. However, to the best of our knowledge, there have been no reports concerning the synthesis of 1,4,5-trisubstituted 5-stibano-1,2,3-triazoles. Herein, we report a novel CuAAC of a simple alkynylstibane, (phenylethynyl)di-*p*-tolylstibane, with organic azides to form fully substituted 5-organostibano-1,2,3-triazoles.

## Results and Discussion

We initially determined the optimal experimental conditions for the cycloaddition of (phenylethynyl)di-*p*-tolylstibane (**1**) with benzylazide (**2a**) under aerobic conditions. Table 1 summarizes the reaction yields obtained with various catalysts and solvents. We first examined the reaction of **1** (0.5 mmol) with **2a** (0.5 mmol) using 5 mol % of various Cu catalysts under aerobic conditions in THF at 60 °C (Table 1, entries 1–9). The results showed that CuBr was the best catalyst and gave the highest yield of the expected 5-stibano-1,2,3-triazole **3a** (Table 1, entry 2). The reaction without the Cu catalyst did not afford **3a** (Table 1, entry 10). Screening of solvents showed that the reaction proceeds effectively in THF (93%) and 1,4-dioxane, whereas DMSO, DMF, EtOH, toluene and 1,2-DCE gave inferior results (Table 1, entries 11–17). The best result was obtained when **1** was treated with **2a** using a catalytic amount of CuBr in THF at 60 °C. This cycloaddition could also be scaled up to 10 mmol and the desired product **3a** was obtained in excellent yields of up to 95%, i.e., 5.11 g of the product could be generated. When Cu(OAc)_2_ was employed as catalyst, 1-benzyl-4-phenyltriazole **4** was isolated in 83% yield as the major product (Table 1, entry 7). Heating of 5-stibano-1,2,3-triazole **3a** in the presence of Cu(OAc)_2_ (5 mol %) under aerobic conditions in THF at 60 °C for 3 h afforded **4** in 91% yield. Furthermore, heating of **1** without **2a** under the same conditions did not give the phenylacetylene and the starting compound was recovered (84%). These two experiments indicate that the formation of 5-*H*-triazole **4** progresses via 5-stibanotriazole **3a**, the cycloaddition product of **1** with **2a**.

**Table 1:** Cu-catalyzed reaction of ethynylstibane **1** with benzylazide **2a**^a^.

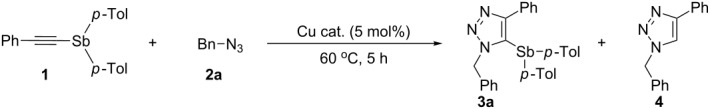				

Entry	Cu cat.	Solvent	Yield [%]^b^	

			**3a**	**4**

1	CuI	THF	76	10
2	CuBr	THF	93	3
3	CuCl	THF	61	19
4	CuOAc	THF	18	30
5	Cu_2_O	THF	25	26
6	CuBr_2_	THF	49	12
7	Cu(OAc)_2_	THF	9	83
8	CuO	THF	9	8
9	CuSO_4_	THF	9	30
10	–	THF	–	2
11	CuBr	1,4-dioxane	61	17
12	CuBr	CH_3_CN	46	42
13	CuBr	DMSO	32	56
14	CuBr	1,2-DCE	18	28
15	CuBr	EtOH	18	14
16	CuBr	DMF	12	83
17	CuBr	toluene	6	3

^a^Reaction conditions: **1** (0.5 mmol), **2a** (0.5 mmol), Cu cat. (0.025 mmol). ^b^Isolated yield.

**Scheme 1:**
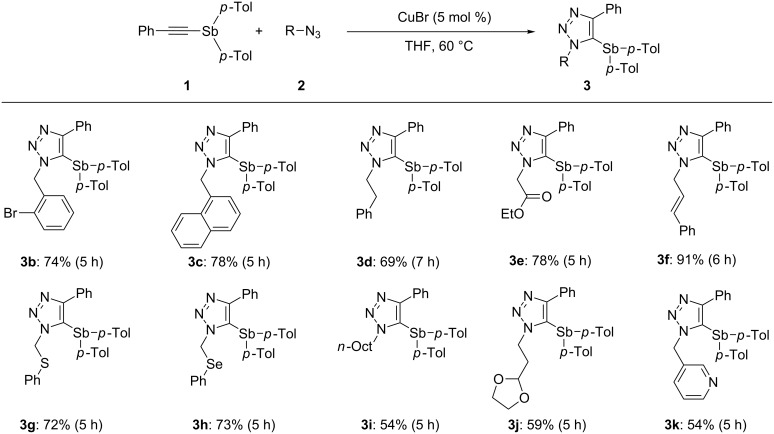
Copper-catalyzed [3 + 2] cycloaddition of **1** with organic azides **2**. Reaction conditions: **1** (0.5 mmol), **2a** (0.5 mmol), Cu cat. (0.025 mmol). Isolated yield are shown.

To investigate the scope and limitations of the CuAAC reaction of stibane, ethynylstibane **1** was reacted with a series of organic azides **2** under optimized conditions (CuBr/THF/60 °C). The results are summarized in Scheme 1. Organic azides having functional groups such as *o*-bromobenzyl, 1-naphthalenemethyl, 2-phenylethyl, (ethoxycarbonyl)methyl, cinnamyl, (phenylthio)methyl and (phenylseleno)methyl afforded the corresponding products **3b–h** in good to excellent yields. In the case of the reaction with *o*-bromobenzyl azide, the carbon–bromine bond of **3b** remained intact, and other byproducts were not observed. Azides containing a linear alkyl group, acetal moiety, and a heteroaromatic ring such as pyridine gave the corresponding triazoles **3i–k** in moderate yields. The CuAAC reaction of **1** with aryl azides such as 4-methylphenyl and 4-cyanophenyl azide gave a complex mixture, presumably due to the steric hindrance introduced by the aryl groups.

**Figure 1:**
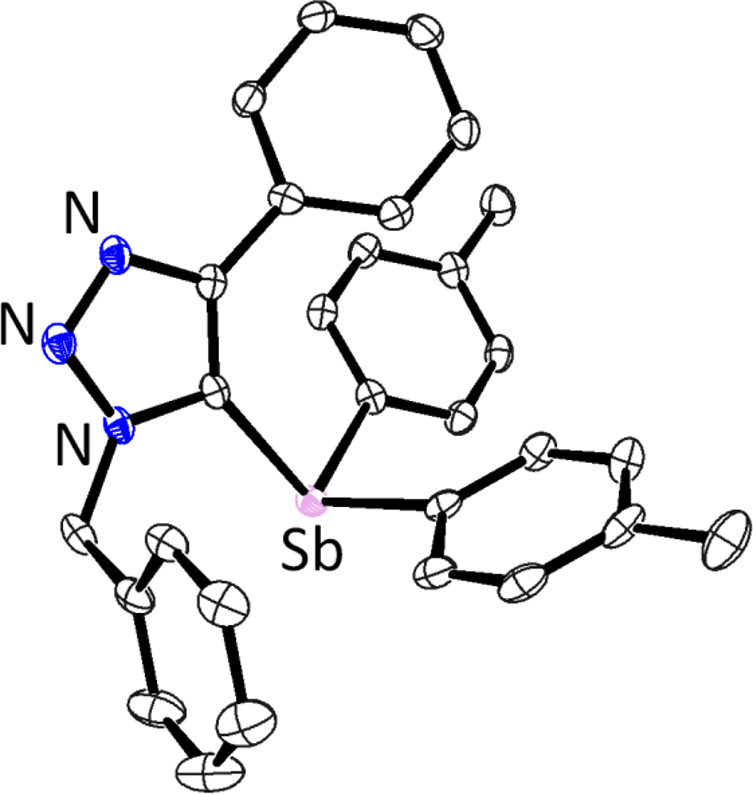
Ortep drawing of **3a** with 50% probability. All hydrogen atoms are omitted for clarity. Two independent molecules exist in the asymmetric unit, one of them is shown.

The regiochemistry of 5-stibanotriazole **3a** was elucidated by ^1^H NMR and confirmed by single-crystal X-ray analysis (Figure 1). A nuclear Overhauser effect (NOE) was observed between the benzyl protons and the aromatic protons of the antimony *p*-tolyl groups. Other triazole **3** showed similar NOE signals between the 1-*N*-substituent protons and the *p*-tolyl protons.

**Scheme 2:**
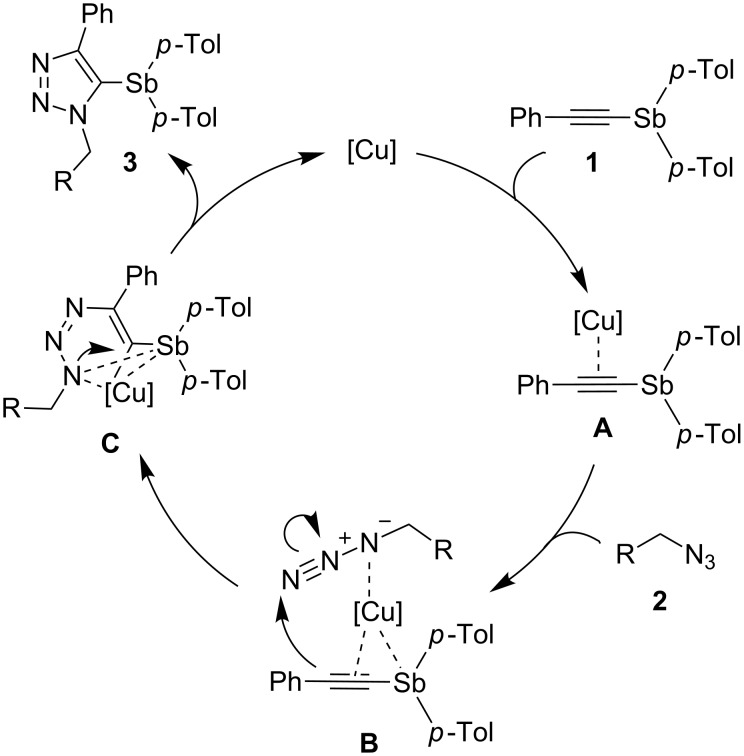
Possible mechanism.

The reaction mechanism of the cyclization is unclear at present. We consider that the catalytic cycle of this reaction would be similar to that for the reaction of 1-iodoalkynes [21,33] and 1-bismuthanoalkynes [24] with organic azides. A possible mechanism of the present Cu-catalyzed cycloaddition is shown in Scheme 2. Initially, π-complex **A** is generated by the reaction of the Cu(I) catalyst and ethynylstibane **1**. Complex **A** coordinates with an organic azide to give complex **B**. Cyclization proceeds via a vinylidene-like transition state **C** to give 5-stibanotriazole **3**.

**Scheme 3:**
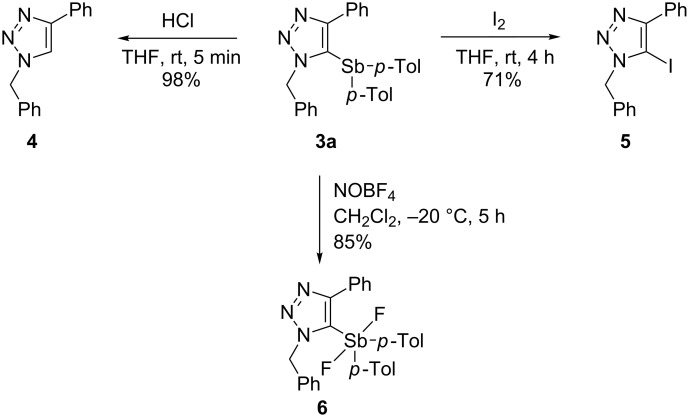
Reaction of **3a** with HCl, I_2_ and NOBF_4_.

To test the reactivity of 5-stibanotriazole **3a** was treated with hydrochloric acid, halogens, and nitrosyl tetrafluoroborate (NOBF_4_) (Scheme 3). The deantimonation of **3a** with HCl gave 1-benzyl-4-phenyl-1,2,3-triazole (**4**) in 98% yield. Iodination of **3a** using I_2_ afforded 5-iodo-4-phenyl-1,2,3-triazole (**5**) in 71% yield. However, the reaction of **3a** with Br_2_ gave a complex mixture. The reaction of **3a** with NOBF_4_ afforded pentavalent organoantimony compound **6** in 85% yield. It is noteworthy that 5-bismuthanotriazole was demetallated upon reaction with NOBF_4_ to give the corresponding 5-nitroso compound [24].

## Conclusion

In conclusion, the Cu-catalyzed azide–alkyne cycloaddition of (phenylethynyl)di-*p*-tolylstibane with organic azides afforded novel 1,4,5-trisubstituted 5-organostibano-1*H*-1,2,3-triazoles, which could be further derivatized using I_2_ and NOBF_4_. Studies on the cycloaddition of diversely-functionalized ethynylstibanes and functionalization at the 5-position of 5-stibanotriazole by electrophilic substitution and cross-coupling reaction are in progress.

## Experimental

**General procedure for the preparation of compounds 3:** CuBr (3.6 mg, 0.025 mmol, 5 mol %), (phenylethynyl)di-*p*-tolylstibane (**1**, 203 mg, 0.5 mmol), and an organic azide (**2**, 0.5 mmol) were dissolved in THF (5 mL). The reaction mixture was stirred at 60 °C and monitored by TLC. Upon disappearance of the starting materials, the reaction mixture was diluted with CH_2_Cl_2_ (30 mL) and water (20 mL). The phases were separated and the aqueous layer was extracted with CH_2_Cl_2_ (20 mL × 2). The combined organic layers were washed 5% aqueous ammonia and water, dried over MgSO_4,_ and concentrated under reduced pressure. The residue was purified by silica gel chromatography (*n*-hexane:AcOEt) to give **3b**, **3i** (8:1), **3f** (6:1), **3g**, **3h** (5:1), **3c**, **3d**, **3e**, **3j** (4:1), **3k** (1:1).

## Supporting Information

File 1 Experimental procedures, full compound characterisation data and X-ray crystallographic data.
