# Supplementary material for: Copper-catalyzed [3 + 2] cycloaddition of (phenylethynyl)di-p-tolylstibane with organic azides
Source: Beilstein J Org Chem. 2016 Jun 23;12:1309–13. doi: 10.3762/bjoc.12.123 (PMC4979910; doi:10.3762/bjoc.12.123)
Supplement: File 1 — Experimental procedures, full compound characterisation data and X-ray crystallographic data. [file Beilstein_J_Org_Chem-12-1309-s001.pdf]

Supporting Information

for

Copper-catalyzed [3 + 2] cycloaddition of (phenylethynyl)di-*p*-tolylstibane with organic azides

Mizuki Yamada[1], Mio Matsumura[1], Yuki Uchida[1], Masatoshi Kawahata[2], Yuki Murata[1], Naoki Kakusawa[3], Kentaro Yamaguchi[2] and Shuji Yasuike*[1]

Address: [1]School of Pharmaceutical Sciences, Aichi Gakuin University, 1-100 Kusumoto-cho, Chikusa-ku, Nagoya 464-8650, Japan, [2]Pharmaceutical Sciences at Kagawa Campus, Tokushima Bunri University, 1314-1 Shido, Sanuki, Kagawa 769-2193, Japan and [3]Faculty of Pharmaceutical Sciences, Hokuriku University, Ho-3 Kanagawa-machi, Kanazawa 920-1181, Japan

Email: Shuji Yasuike* - s-yasuik@dpc.agu.ac.jp

*Corresponding author

Experimental procedures, full compound characterisation data and X-ray crystallographic data

Table of contents

1, General information S2

2, Synthesis and characterization of (phenylethynyl)di-*p*-tolylstibane (**1**) S2

3, Characterization of 5-stibanotriazoles (**3**) S3

4, Reaction of **3a** with HCl, I_2 and $NOBF_4$ S6

5, X-ray crystallographic data S7

6, Copies of ^{1}H and ^{13}C NMR spectra S9

7, References S24

General information

Melting points were measured on a Yanagimoto micro melting point hot-stage apparatus (MP-S3) and reported as uncorrected values. ^{1}H NMR (TMS: δ: 0.00 ppm as an internal standard) and ^{13}C NMR ($CDCl_3$: δ: 77.00 ppm as an internal standard) and ^{19}F NMR (CCl_3F : 0.00 ppm as an extremal standard) spectra were recorded on JEOL JNM-AL400 (400 MHz and 100 MHz) and JNM-ECZ400S (400 MHz, and 376 MHz) spectrometers in $CDCl_3$. Mass spectra were obtained on a JEOL JMP-DX300 instrument (70 eV, 300 μA). IR spectra were recorded on a Shimadzu FTIR-8400S spectrophotometer and reported in terms of frequency of absorption (cm^{-1}). Only selected IR bands are reported. Chromatographic separations were carried out using Silica Gel 60N (Kanto Chemical Co., Inc.). Thin-layer chromatography (TLC) was performed using Merck Pre-coated TLC plates (silica gel 60 F_{254}). Benzyl azide and azidomethyl phenyl sulfide were purchased from Wako Pure Chemical Industries and Sigma-Aldrich, respectively. Other azide compounds were prepared according to the published procedures (**2b** [1], **2c** [2], **2d**, **2i**, **2j** [3], **2e, 2f** [4], **2h** [5, 6], **2k** [7]).

Synthesis and characterization of (phenylethynyl)di-*p*-tolylstibane (1)

(phenylethynyl)di-*p*-tolylstibane (**1**) were prepared according to the reported procedure [8]. An ether solution (50 mL) of di-*p*-tolylantimony(III) bromide, synthesized from redistribution of tri-*p*-tolylstibane (11.06 g, 28 mmol) and tribromoantimony (5.06 g, 14 mmol) was added dropwise at 0 ºC to an ether solution (50 mL) of lithium acetylide, prepared from the appropriate phenylacetylene (42 mmol) and butyllithium (1.54 M solution in hexane, 42 mmol). After stirring the mixture at the same temperature for 2 h, the reaction mixture was diluted with ether (50 mL) and quenched with water. The reaction mixture was separated and the aqueous layer was extracted with ether (50 mL). The combined organic layer was dried over anhydrous $MgSO_4$, and concentrated under reduced pressure. The residue was purified by silica gel chromatography (*n*-hexane/CH_2Cl_2 = 5:1), affording compound **1** as colorless needles (11.56 g, 68% yield), mp 58-61 °C (from ether-methanol). ^{1}H NMR (400 MHz, $CDCl_3$) δ: 2.32 (6H, s), 7.16 (4H, d, J = 7.8 Hz), 7.28-7.31 (3H, m), 7.48-7.52 (2H, m), 7.61 (4H, d, J = 7.8 Hz). ^{13}C NMR (100 MHz, $CDCl_3$) δ: 21.4 (q), 86.0 (s), 110.9 (s), 123.4 (s), 128.2 (d), 128.4 (d), 129.8 (d), 132.0 (d), 134.4 (s), 135.5 (d), 138.7 (s). FTIR (KBr): 2363 cm^{-1}. LRMS (EI) m/z: 405 (M^+, 75), 327 (10), 303 (45), 212 (100), 77 (20). Anal. Calc. for $C_{22}H_{19}Sb$: C, 65.22; H, 4.73. Found: C, 65.19; H, 4.58.

Characterization of 5-stibanotriazoles (3)

1-Benzyl-5-(di-*p*-tolylstibano)-4-phenyl-1*H*-1,2,3-triazole (3a)

Colorless prisms (250 mg, 93% yield), mp 128-129 ºC (from *n*-hexane-CH_2Cl_2). ^{1}H NMR (400 MHz, $CDCl_3$) δ: 2.29 (6H, s), 5.32 (2H, s), 6.75 (2H, d, J = 7.3 Hz), 7.01 (4H, d, J = 8.3 Hz), 7.10-7.23 (10H, m), 7.45 (2H, m). ^{13}C NMR (100 MHz, $CDCl_3$) δ: 21.3 (q), 53.9 (t), 126.8 (s), 127.0 (d), 127.6 (d), 127.7 (d), 127.9 (d), 128.3 (d), 129.0 (d), 129.9 (d), 130.7 (s), 131.9 (s), 135.6 (s), 135.7 (d), 139.1 (s), 156.5 (s). LRMS (FAB) m/z: 538 (M^+, 40), 303 (12), 235 (25), 206 (100), 182 (82), 149 (50), 116 (70), 91 (40), 89 (25), 65 (20). Anal. Calc. for $C_{29}H_{26}N_3Sb$: C, 64.71; H, 4.87; N, 7.81. Found: C, 65.11; H, 4.88; N, 7.89.

1-(2-Bromobenzyl)-5-(di-*p*-tolylstibano)-4-phenyl-1*H*-1,2,3-triazole (3b)

Colorless plates (228 mg, 74% yield), mp 144-145 ºC (from *n*-hexane-CH_2Cl_2). ^{1}H NMR (400 MHz, $CDCl_3$) δ: 2.27 (6H, s), 5.31 (2H, s), 6.11-6.13 (1H, m), 6.98 (4H, d, J = 7.3 Hz), 7.01-7.07 (2H, m), 7.14 (4H, d, J = 7.3 Hz), 7.29-7.36 (4H, m), 7.62-7.65 (2H, m). ^{13}C NMR (100 MHz, $CDCl_3$) δ: 21.3 (q), 54.3 (t), 121.5 (s), 127.1 (d), 127.3 x 2 (s, d), 128.1 (d), 128.3 (d), 128.6 (d), 129.0 (d), 130.05 (d), 130.09 (s), 132.0 x 2 (s, d), 135.6 x 2 (s, d), 139.3 (s), 156.6 (s). LRMS (FAB) m/z: 618 (M^+, 100), 303 (17), 236 (11), 206 (17), 169 (20), 91 (10). Anal. Calc. for $C_{29}H_{25}BrN_3Sb$: C, 56.43; H, 4.08; N, 6.81. Found: C, 56.58; H, 4.12; N, 6.97.

5-(Di-*p*-tolylstibano)-1-(naphthalen-1-ylmethyl)-4-phenyl-1*H*-1,2,3-triazole (3c)

Colorless prisms (229 mg, 78% yield), mp 156-157 ºC (from *n*-hexane-CH_2Cl_2). ^{1}H NMR (400 MHz, $CDCl_3$) δ: 2.13 (6H, s), 5.79 (2H, s), 6.42 (1H, d, J = 7.3 Hz), 6.80 (4H, d, J = 7.8 Hz), 7.04 (4H, d, J = 7.8 Hz), 7.20-7.32 (4H, m), 7.35 (2H, d, J = 6.0 Hz), 7.41-7.46 (1H, m), 7.58-7.62 (2H, m), 7.68 (1H, d, J = 8.3 Hz), 7.78 (1H, d, J = 8.3 Hz). ^{13}C NMR (100 MHz, $CDCl_3$) δ: 21.2 (q), 52.2 (t), 122.3 (d), 123.7 (d), 125.1 (d), 125.7 (d), 125.9 (d), 127.2 (s), 127.9 (d), 128.0 (d), 128.2 (d), 128.5 (d), 128.9 (d), 129.8 (d), 130.0 (s), 130.5 (s), 131.9 (s), 132.1 (s), 133.2 (s), 135.6 (d), 139.0 (s), 156.5 (s). LRMS (FAB) m/z: 588 (M^+, 60), 303 (16), 182 (12), 141 (100), 91 (10). Anal. Calc. for $C_{33}H_{28}N_3Sb$: C, 67.37; H, 4.80; N, 7.14. Found: C, 67.05; H, 4.79; N, 7.15.

5-(Di-*p*-tolylstibano)-1-phenethyl-4-phenyl-1*H*-1,2,3-triazole (3d)

Colorless prisms (191 mg, 69% yield), mp 136-137 ºC (from *n*-hexane-CH_2Cl_2). ^{1}H NMR (400 MHz, $CDCl_3$) δ: 2.33 (6H, s), 2.93 (2H, t, J = 8.3 Hz), 4.21 (2H, t, J = 8.3 Hz), 6.75 (2H, dd, J = 7.3 Hz, 3.9 Hz), 7.13 (4H, d, J = 7.8 Hz), 7.18-7.19 (3H, m), 7.26-7.30 (7H, m), 7.53-7.56 (2H, m). ^{13}C NMR (100 MHz, $CDCl_3$) δ: 21.3 (q), 36.7 (t), 51.8 (t), 126.6 (d), 126.7 (s), 127.9 (d), 128.2 (d), 128.3 (d), 128.7 (d), 129.1 (d), 130.3 (d), 130.9 (s), 132.1 (s), 135.7 (d), 137.3 (s), 139.4 (s), 156.2 (s). LRMS (FAB) m/z: 552 (M^+, 100), 303 (10), 105 (40), 91 (10). Anal. Calc. for $C_{30}H_{28}N_3Sb$: C, 65.24; H, 5.11; N, 7.61. Found: C, 65.51; H, 5.20; N, 7.69.

Ethyl 2-[5-(di-*p*-tolylstibano)-4-phenyl-1*H*-1,2,3-triazol-1-yl]acetate (3e)

Colorless prisms (208 mg, 78% yield), mp 92-94 ºC (from *n*-hexane-CH_2Cl_2). ^{1}H NMR (400 MHz, $CDCl_3$) δ: 1.13 (3H, t, J = 7.0 Hz), 2.34 (6H, s), 3.92 (2H, q, J = 7.0 Hz), 4.78 (2H, s), 7.14 (4H, d, J = 8.3 Hz), 7.27-7.35 (7H, m), 7.60-7.63 (2H, m). ^{13}C NMR (100 MHz, $CDCl_3$) δ: 13.8 (q), 21.4 (q), 51.4 (t), 61.7 (t), 127.6 (s), 128.1 (d), 128.3 (d), 128.9 (d), 130.2 (d), 130.8 (s), 132.0 (s), 136.0 (d), 139.5 (s), 156.2 (s), 166.6 (s). FTIR (KBr): 1751 cm^{-1}. LRMS (FAB) m/z: 534 (M^+, 40), 303 (15), 241 (30), 185 (70), 149 (80), 93 (100), 75 (90). Anal. Calc. for $C_{26}H_{26}N_3O_2Sb$: C, 58.45; H, 4.91; N, 7.87. Found: C, 58.31; H, 4.88; N, 7.87.

1-Cinnamyl-5-(di-*p*-tolylstibano)-4-phenyl-1*H*-1,2,3-triazole (3f)

Colorless prisms (257 mg, 91% yield), mp 139-142 ºC (from *n*-hexane-CH_2Cl_2). ^{1}H NMR (400 MHz, $CDCl_3$) δ: 2.31 (6H, s), 4.80 (2H, dd, J = 6.3 Hz, 1.5 Hz), 5.73 (1H, dt, J = 16.1 Hz, 6.3 Hz), 6.05 (1H, d, J = 16.1 Hz), 7.08-7.13 (6H, m), 7.21-7.31 (10H, m), 7.56-7.60 (2H, m). ^{13}C NMR (100 MHz, $CDCl_3$) δ: 21.3 (q), 52.6 (t), 123.3 (d), 126.5 x 2 (s, d), 127.85 (d), 127.93 (d), 128.2 (d), 128.4 (d), 129.0 (d), 130.2 (d), 130.8 (s), 132.1 (s), 133.4 (d), 135.8 (d), 135.9 (s), 139.4 (s), 156.7 (s). LRMS (FAB) m/z: 564 (M^+, 27), 303 (12), 297 (11), 241 (45), 221 (10), 185 (85), 149 (95), 93 (100), 75 (90), 57 (47), 45 (23), 31 (10). Anal. Calc. for $C_{31}H_{28}N_3Sb$: C, 65.98; H, 5.00; N, 7.45. Found: C, 65.72; H, 4.89; N, 7.43.

5-(Di-*p*-tolylstibano)-4-phenyl-1-[(phenylsulfanyl)methyl]-1*H*-1,2,3-triazole (3g)

Colorless prisms (205 mg, 72% yield), mp 73-74 ºC (from *n*-hexane-AcOEt). ^{1}H NMR (400 MHz, $CDCl_3$) δ: 2.30 (6H, s), 5.22 (2H, s), 7.07 (4H, d, J = 7.8 Hz), 7.16-7.46 (10H, m), 7.34 (4H, d, J = 7.8 Hz). ^{13}C NMR (100 MHz, $CDCl_3$) δ: 21.3 (q), 54.2 (t), 126.2 (s), 127.8 (d), 128.0 (d), 128.3 (d), 128.6 (d), 129.1 (d), 130.0 (d), 131.3 (s), 131.8 (s), 131.9 (s), 132.1 (d), 136.1 (d), 139.4 (s), 156.2 (s). LRMS (FAB) m/z: 570 (M^+, 100), 303 (46), 236 (15), 182 (17), 123 (68), 91 (10). Anal. Calc. for $C_{29}H_{26}N_3SSb$: C, 61.07; H, 4.59; N, 7.37. Found: C, 61.16; H, 5.04; N, 7.42.

5-(Di-*p*-tolylstibano)-4-phenyl-1-[(phenylselanyl)methyl]-1*H*-1,2,3-triazole (3h)

Colorless prisms (225 mg, 73% yield), mp 93-94 ºC (from *n*-hexane-AcOEt). ^{1}H NMR (400 MHz, $CDCl_3$) δ: 2.32 (6H, s), 5.32 (2H, s), 7.11 (4H, d, J = 7.3 Hz), 7.19-7.29 (6H, m), 7.34 (4H, d, J = 7.3 Hz), 7.37-7.39, (2H, m), 7.47-7.51 (2H, m). ^{13}C NMR (100 MHz, $CDCl_3$) δ: 21.4 (q), 46.0 (t), 126.4 (s), 127.85 (s), 127.87 (d), 128.1 (d), 128.5 (d), 128.6 (d), 129.2 (d), 130.1 (d), 131.1 (s), 131.9 (s), 134.4 (d), 136.1 (d), 139.4 (s), 156.3 (s). LRMS (FAB) m/z: 618 ($M+1^+$, 100), 462 (16), 369 (15), 303 (72), 236 (18), 182 (30), 105 (28), 91 (30). Anal. Calc. for $C_{29}H_{26}N_3SbSe$: C, 56.43; H, 4.25; N, 6.81. Found: C, 56.55; H, 4.36; N, 6.87.

5-(Di-*p*-tolylstibano)-1-octyl-4-phenyl-1*H*-1,2,3-triazole (3i)

Colorless oil (151 mg, 54% yield), ^{1}H NMR (400 MHz, $CDCl_3$) δ: 0.85-0.89 (5H, m), 0.98-1.05 (2H,

m), 1.07-1.21 (4H, m), 1.22-1.27 (2H, m), 1.38-1.46 (2H, m), 2.35 (6H, s), 3.93 (2H, t, *J* = 7.8 Hz), 7.16 (4H, d, *J* = 7.8 Hz), 7.28-7.34 (7H, m), 7.56-7.59 (2H, m). ^{13}C NMR (100 MHz, $CDCl_3$) δ: 14.1 (q), 21.3 (q), 22.6 (t), 26.4 (t), 29.01 (t), 29.04 (t), 30.9 (t), 31.7 (t), 51.1 (t), 126.1 (s), 127.9 (d), 128.2 (d), 129.0 (d), 130.2 (d), 130.9 (s), 132.2 (s), 135.7 (d), 139.4 (s), 156.3 (s). LRMS (FAB) m/z: 560 (M^+, 100), 288 (20), 236 (12), 182 (15), 119 (40), 85 (38), 77 (15). HRMS (FAB) m/z: 559.1935 (Calc. for $C_{30}H_{36}N_3Sb$: 559.1947).

1-[2-(1, 3-Dioxolan-2-yl)ethyl]-5-(di-*p*-tolylstibano)-4-phenyl-1*H*-1,2,3-triazole (3j)

Colorless plates (162 mg, 59% yield), mp 130-132 ºC (from *n*-hexane-CH_2Cl_2). ^{1}H NMR (400 MHz, $CDCl_3$) δ: 1.86-1.91 (2H, m), 2.34 (6H, s), 3.69-3.84 (4H, m), 4.10-4.14 (2H, m), 4.60 (1H, t, *J* = 4.6 Hz), 7.14 (4H, d, *J* = 7.8 Hz), 7.27-7.30 (7H, m), 7.52-7.55 (2H, m). ^{13}C NMR (100 MHz, $CDCl_3$) δ: 21.3 (q), 34.5 (t), 46.4 (t), 64.8 (t), 101.8 (d), 126.5 (s), 127.9 (d), 128.2 (d), 129.1 (d), 130.2 (d), 130.9 (s), 132.1 (s), 135.8 (d), 139.4 (s), 156.3 (s). LRMS (FAB) m/z: 548 (M^+, 100), 303 (9), 182 (8). Anal. Calc. for $C_{27}H_{28}N_3O_2Sb$: C, 59.15; H, 5.15; N, 7.66. Found: C, 59.73; H, 5.22; N, 7.71.

3-{[5-(Di-*p*-tolylstibano)-4-phenyl-1*H*-1,2,3-triazol-1-yl]methyl}pyridine (3k)

Colorless prisms (146 mg, 54% yield), mp 101-102 ºC (from *n*-hexane-CH_2Cl_2). ^{1}H NMR (400 MHz, $CDCl_3$) δ: 2.32 (6H, s), 5.27 (2H, s), 7.02-7.09 (6H, m), 7.18 (4H, d, *J* = 7.8 Hz), 7.27-7.34 (3H, m), 7.55-7.58 (2H, m), 7.92 (1H, s), 8.43 (1H, br). ^{13}C NMR (100 MHz, $CDCl_3$) δ: 21.3 (q), 51.4 (t), 123.2 (d), 126.9 (s), 128.1 (d), 128.2 (d), 129.0 (d), 130.2 (d), 130.3 (s), 131.4 (s), 131.8 (s), 134.6 (d), 135.7 (d), 139.6 (s), 148.1 (d), 148.8 (d), 157.0 (s). LRMS (FAB) m/z: 539 (M^+, 100), 303 (18), 207 (30), 206 (20), 182 (19), 119 (15), 93 (62), 92 (22). Anal. Calc. for $C_{28}H_{25}N_4Sb$: C, 62.36; H, 4.67; N, 10.39. Found. C, 62.51; H, 4.87; N, 10.42.

Reaction of 3a with HCl, I_2 and $NOBF_4$

1-Benzyl-4-phenyl-1*H*-1,2,3-triazole (4) [9]

1-Benzyl-5-(di-*p*-tolylstibano)-4-phenyl-1*H*-1,2,3-triazole (**3a**) (0.25 mmol) and 10% HCl (8 mL) in THF (8 mL) was stirred at room temperature for 5 min. After dilution with water (20 mL), the mixture was extracted with Et_2O (20 mL × 2). The organic layer was collected, dried over $MgSO_4$, and concentrated under reduced pressure. The residue was purified by silica gel column chromatography (*n*-hexane/AcOEt = 4:1), affording compound **4** as colorless needles (58 mg, 98% yield), mp 126 °C (from *n*-hexane–CH_2Cl_2). ^{1}H NMR (400 MHz, $CDCl_3$) δ: 5.58 (2H, s), 7.31-7.42 (8H, m), 7.66 (1H, s), 7.80 (2H, d, J = 7.8 Hz). ^{13}C NMR (100 MHz, $CDCl_3$) δ: 54.1 (t), 119.4 (d), 125.6 (d), 127.9 (d), 128.1 (d), 128.6 (d), 128.7 (d), 129.0 (d), 130.4 (s), 134.6 (s), 148.1 (s). LRMS (EI) m/z: 235 (M^+, 13), 206 (60), 180 (11), 116 (100), 91 (87), 65 (20). Anal. Calc. for $C_{15}H_{13}N_3$: C, 76.57; H, 5.57; N, 17.86. Found: C, 76.77; H, 5.69; N, 17.64.

1-Benzyl-5-iodo-4-phenyl-1*H*-1,2,3-triazole (5) [9]

To a solution of 1-benzyl-5-(di-*p*-tolylstibano)-4-phenyl-1*H*-1,2,3-triazole (**3a**, 269 mg, 0.5 mmol) in THF (3 mL) was added a solution of iodine (140 mg, 0.55 mmol) in THF (3 mL) at 0 °C. Then reaction mixture was stirred at room temperature for 4 h. The reaction mixture was diluted with ethyl acetate (30 mL) and washed with aqueous $Na_2S_2O_3$. The organic layer was collected, dried over $MgSO_4$, and concentrated under reduced pressure. The residue was purified by silica gel column chromatography (*n*-hexane/AcOEt = 4:1), affording compound **5** as colorless prisms (128 mg, 71% yield), mp 137-138 °C (from *n*-hexane–CH_2Cl_2). ^{1}H NMR (400 MHz, $CDCl_3$) δ: 5.68 (2H, s), 7.30-7.48 (8H, m), 7.94 (2H, d, J = 7.3 Hz). ^{13}C NMR (100 MHz, $CDCl_3$) δ: 54.4 (t), 76.4 (s), 127.4 (d), 127.8 (d), 128.5 (d), 128.5 (d), 128.6 (d), 128.9 (d), 130.2 (s), 134.3 (s), 150.2 (s). LRMS (EI) m/z: 361 (M^+, 13), 234 (22), 206 (95), 179 (27), 115 (11), 91 (100), 65 (14). Anal. Calc. for $C_{15}H_{12}N_3$: C, 49.88; H, 3.35; N, 11.63. Found: C, 50.12; H, 3.54; N, 11.77.

(1-Benzyl-4-phenyl-1*H*-1,2,3-triazol-5-yl) difluorodi-*p*-tolylstibane (6)

To a solution of 1-benzyl-5-(di-*p*-tolylstibano)-4-phenyl-1*H*-1,2,3-triazole (**3a**, 269 mg, 0.5 mmol) in CH_2Cl_2 (3 mL) was added a solution of nitrosyl tetrafluoroborate (177 mg, 1.5 mmol) in THF (3 mL) at –20 °C. The mixture was stirred at –20 °C for 1 h; then, the temperature was allowed to increase to 0 °C, and the mixture was stirred for 4 h. The reaction mixture was diluted with CH_2Cl_2 (30 mL) and washed with water. The organic layer was collected, dried over $MgSO_4$, and concentrated under reduced pressure. The residue was purified by silica gel chromatography (*n*-hexane/AcOEt = 3:1) to obtain, compound **6** as a colorless oil (244 mg, 85% yield). ^{1}H NMR (400 MHz, $CDCl_3$) δ: 2.32 (6H, s), 5.70 (2H, s), 6.69 (2H, d, J = 7.3 Hz), 6.98-7.20 (10H, m), 7.45 (2H, d, J = 7.3 Hz), 7.66 (4H, d, J = 8.3 Hz). ^{13}C NMR (100 MHz, $CDCl_3$) δ: 21.5 (q), 54.4 (t), 126.5

(d), 127.7 (d), 127.9 x 2 (s, d), 128.4 (d), 128.6 (d), 129.4 (d), 130.2 (s), 130.6 (d), 134.9 (s), 135.1 (d), 143.4 (s), 154.5 (s). ^{19}F NMR (376 MHz, $CDCl_3$) δ: -135.2. LRMS (FAB) m/z: 576 (M^+, 68), 583 (28), 466 (16), 288 (50), 206 (35), 154 (86), 119 (100), 91 (85), 85 (85), 39 (20). HRMS (FAB) m/z: 575.1144 (Calc. for $C_{29}H_{26}F_2N_3Sb$: 575.1133).

X-ray crystallographic data

The X-ray diffraction measurements of compounds **3a** were carried out using a 100 K Bruker APEX II CCD area-detector diffractometer using Mo Kα radiation (λ = 0.71073 Å). The SADABS software was used for absorption correction. The structure was solved by direct methods using SHELXS-97 [10] followed by successive refinements using the full-matrix least-squares method on F^2 using SHELXL-2014 [11]. All the nonhydrogen atoms were refined anisotropically, whereas the hydrogen atoms were refined isotropically.

Crystal data of **3a**
$C_{29}H_{26}N_3Sb$, M = 538.28, 0.19 x 0.18 x 0.18 mm, monoclinic $P21/n$, Z = 8, D_{calc} = 1.472 Mg/m^3, a = 13.6590(12), b = 20.2294(18), c = 17.5830(16) Å, β = 91.1810(10)°, V = 4857.4(8) Å^3, The final R_1 and wR_2 were 0.0298 and 0.0677 ($I > 2\sigma(I)$).
The experimental and refinement details of the X-ray crystallographic structures of compound can be obtained free of charge from the Cambridge Crystallographic Data Centre (http://www.ccdc.cam.ac.uk), CCDC 1470151.

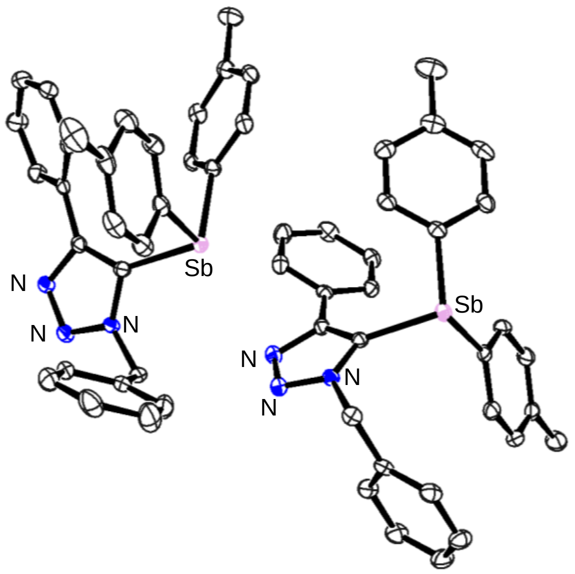

Figure S1: ORTEP drawing of compound **3a** with 50% probability (two independent molecules in the asymmetric unit). All hydrogen atoms are omitted for clarity.

Copies of ^{1}H and ^{13}C NMR spectra

^{1}H NMR of **1**

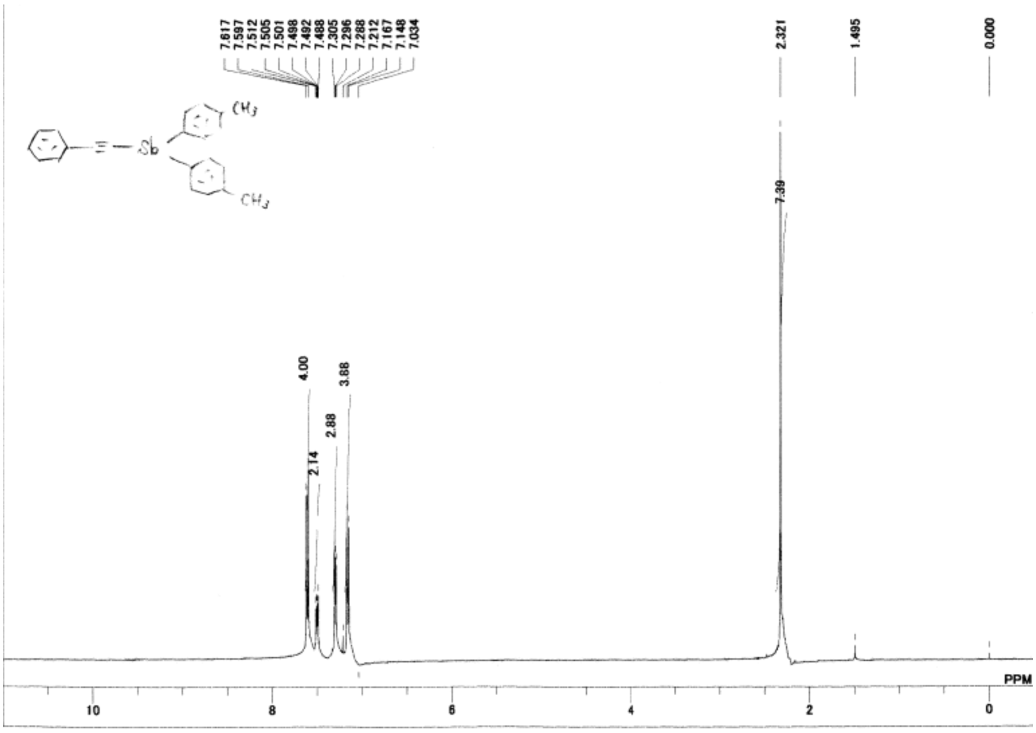

^{13}C NMR of **1**

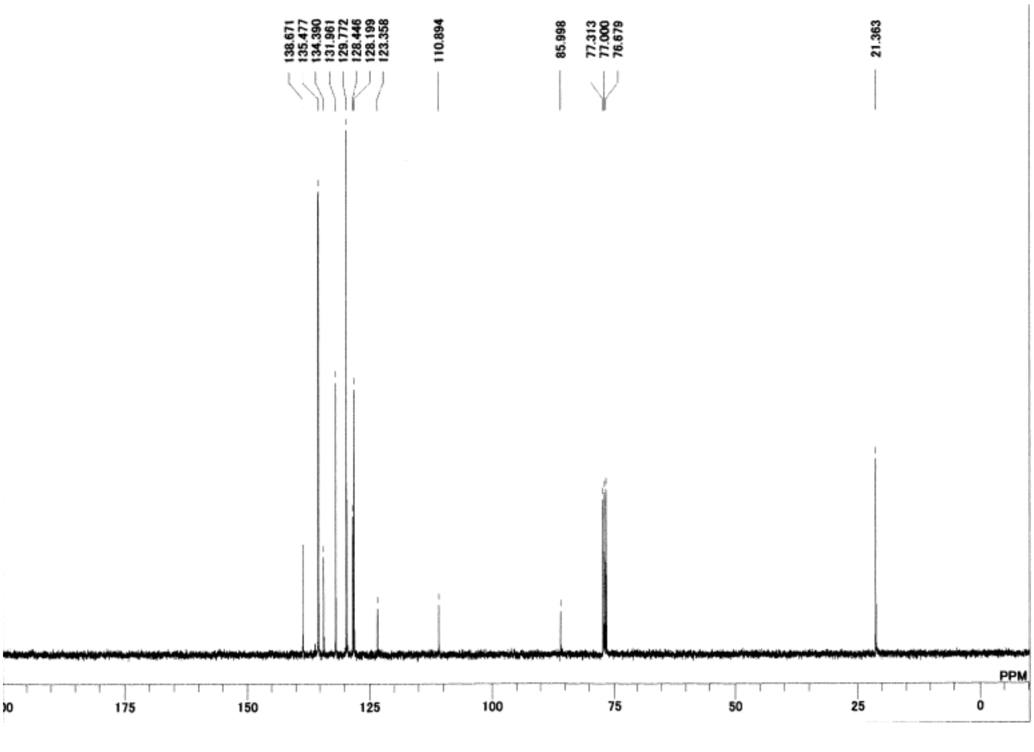

^{1}H NMR of **3a**

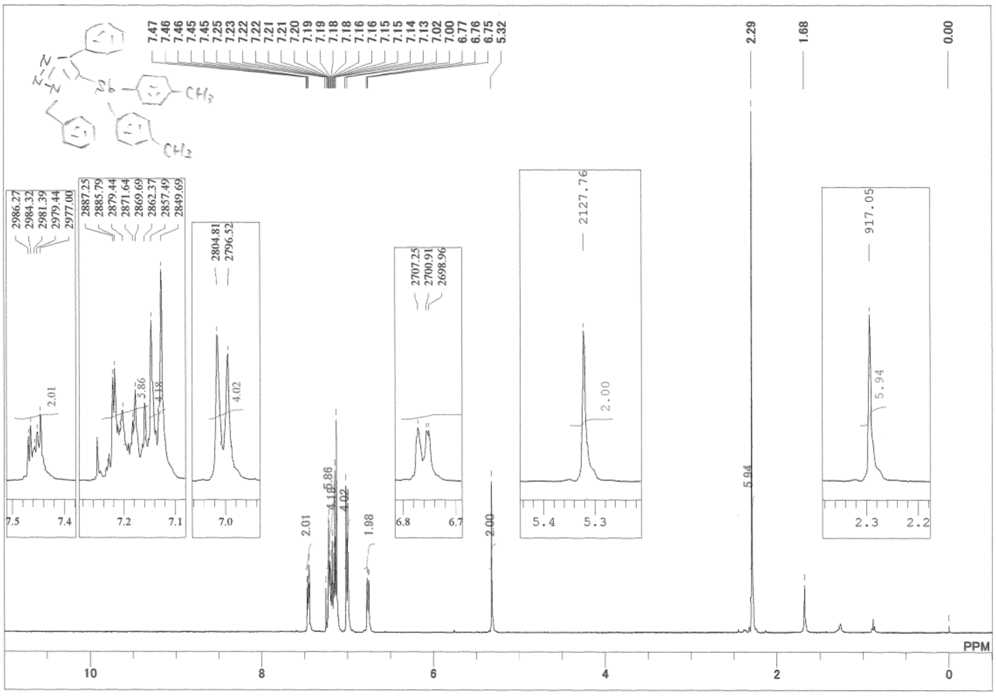

^{13}C NMR of **3a**

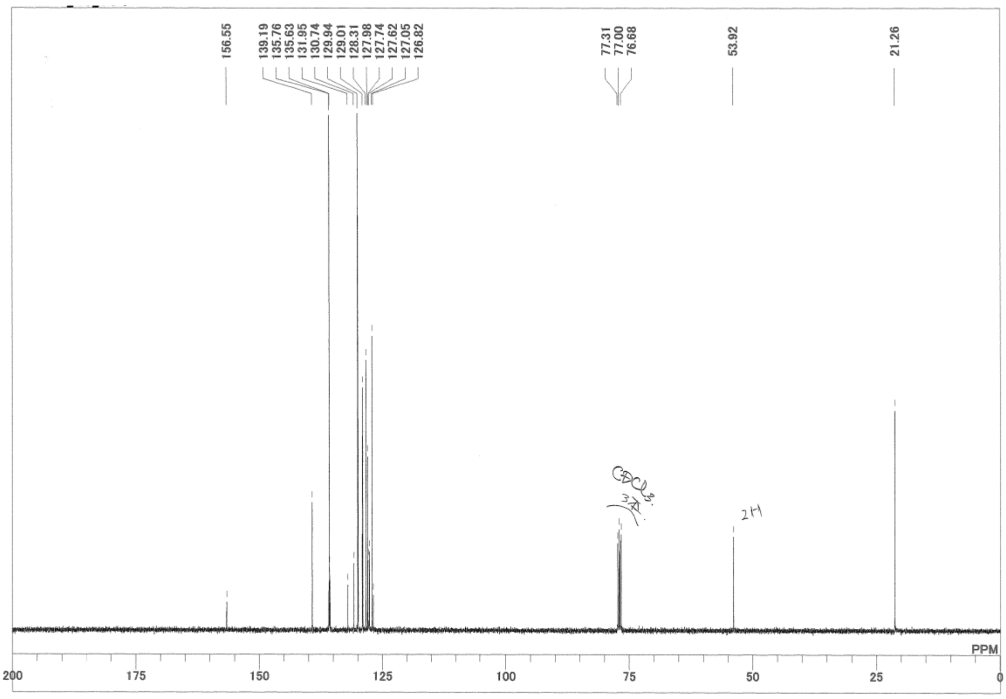

^{1}H NMR of **3b**

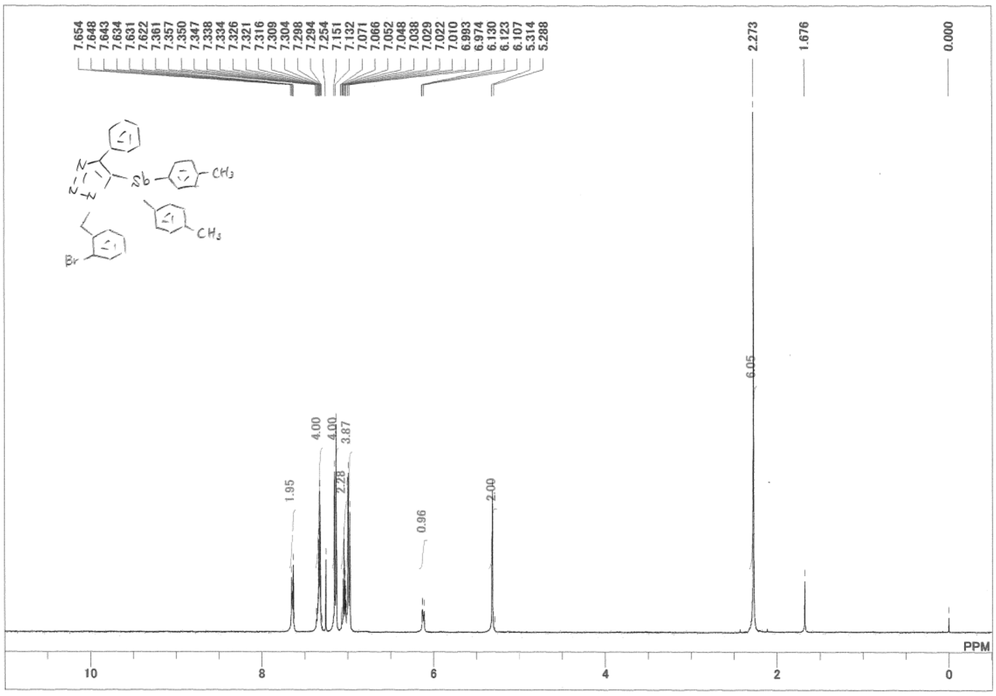

^{13}C NMR of **3b**

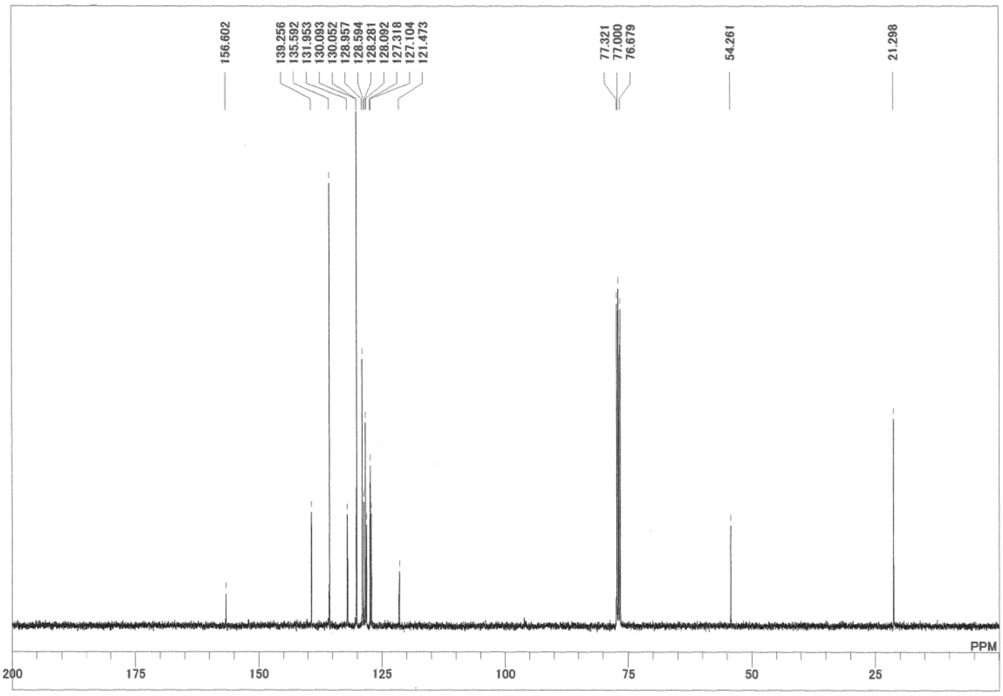

^{1}H NMR of **3c**

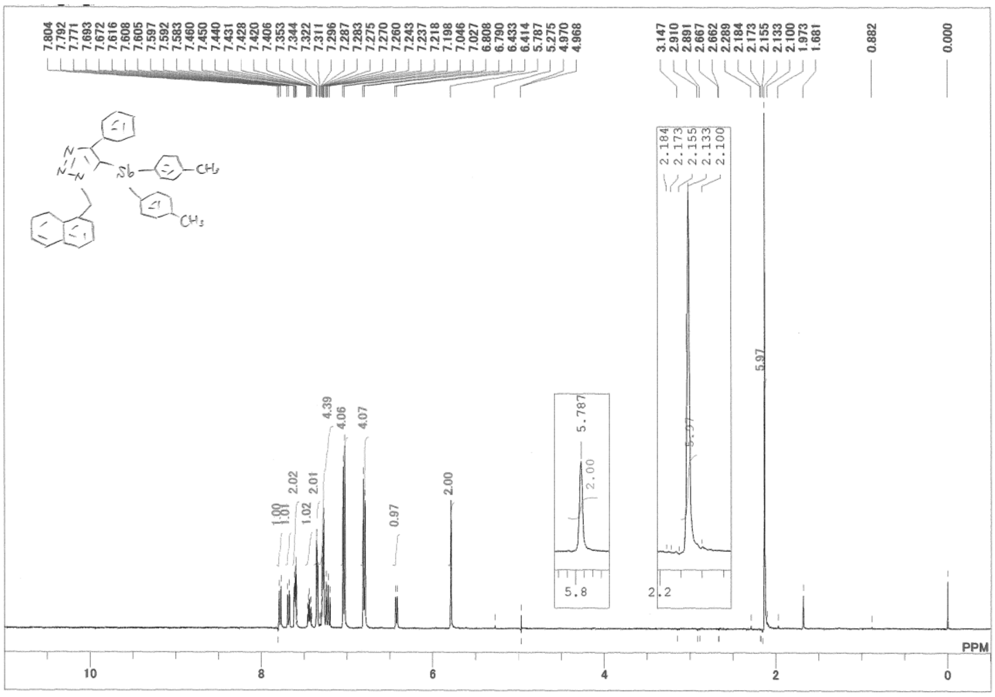

^{13}C NMR of **3c**

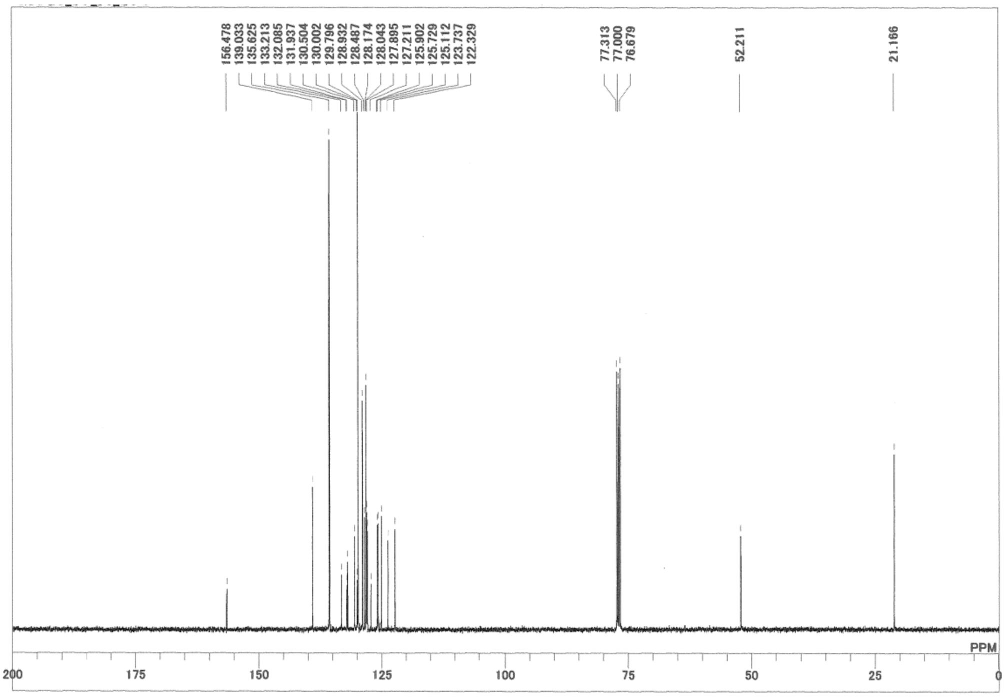

^{1}H NMR of **3d**

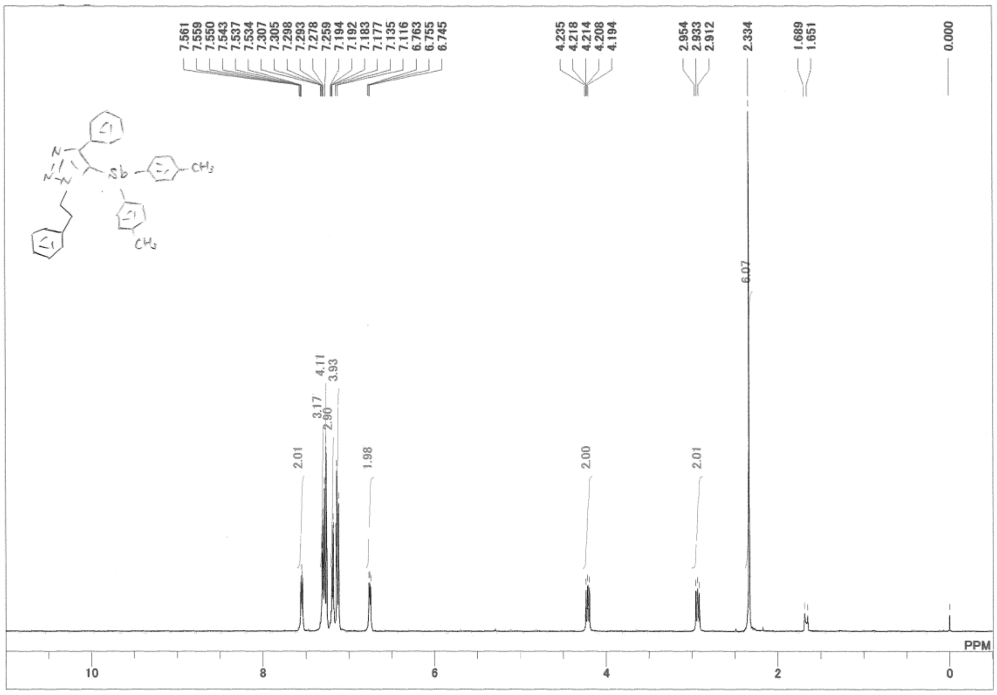

^{13}C NMR of **3d**

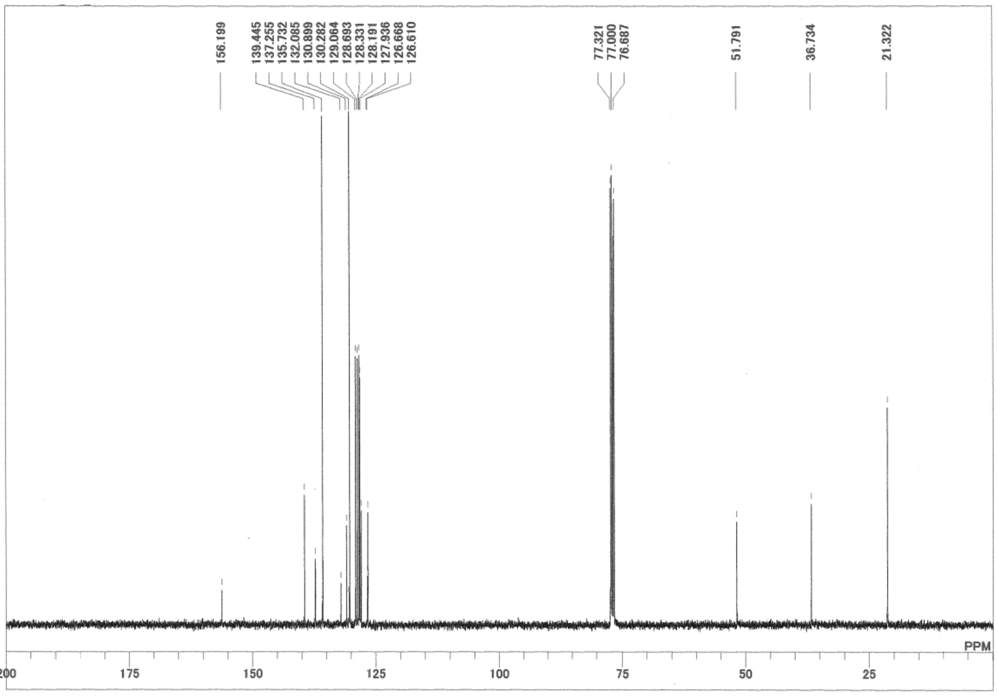

^{1}H NMR of **3e**

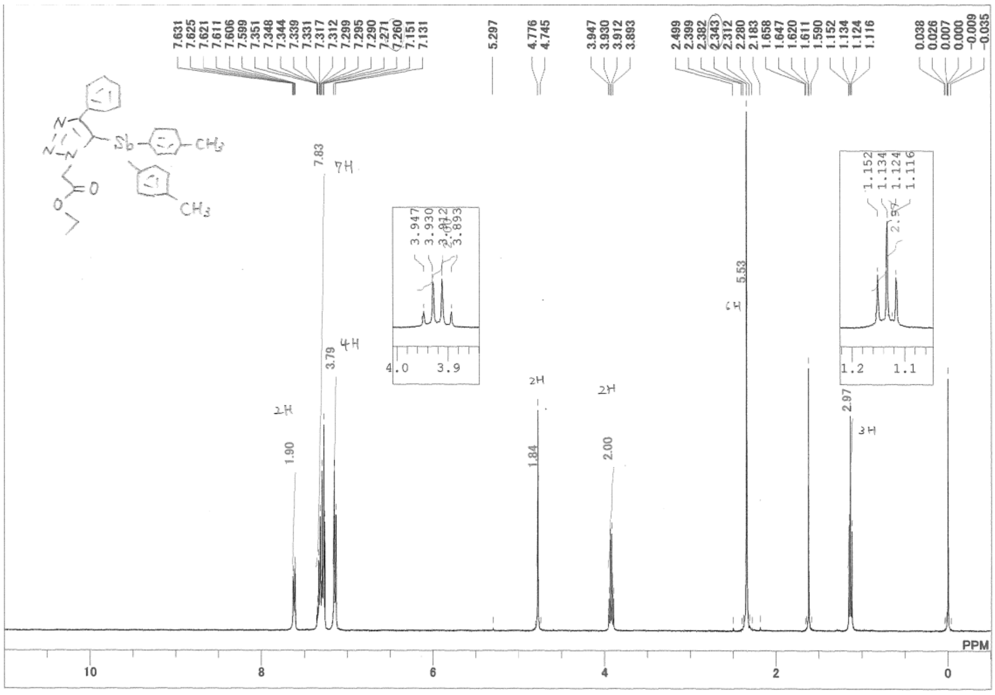

^{13}C NMR of **3e**

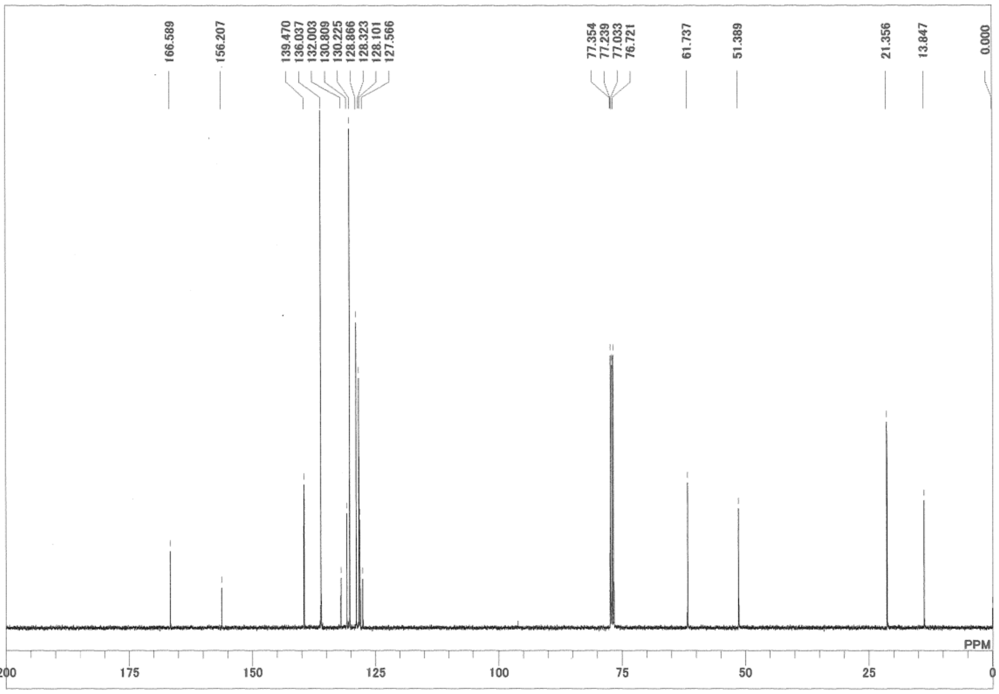

^{1}H NMR of **3f**

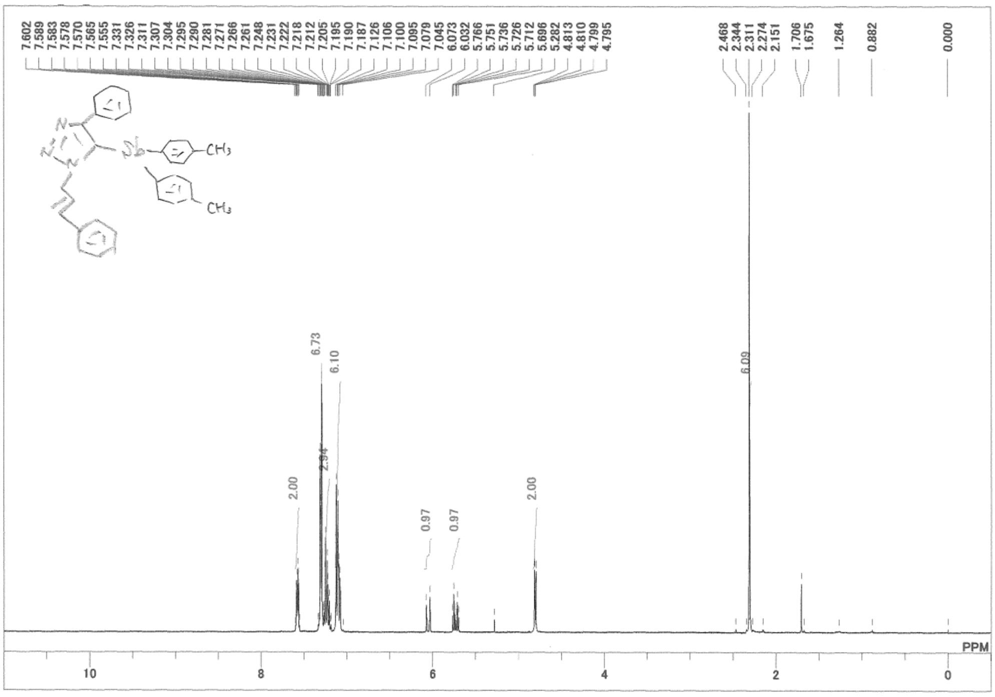

^{13}C NMR of **3f**

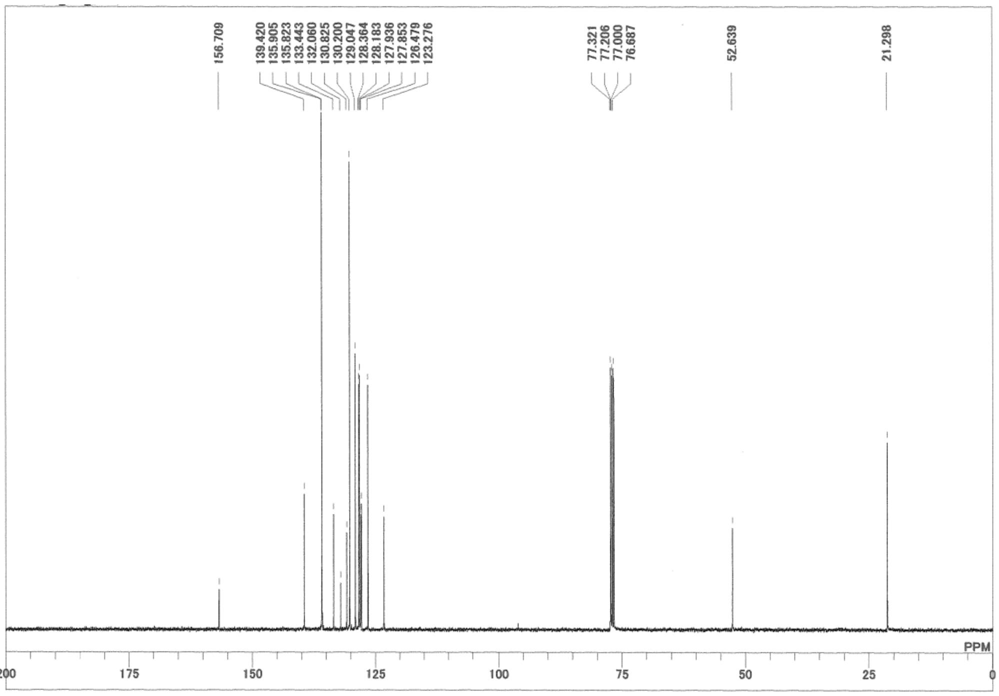

^{1}H NMR of **3g**

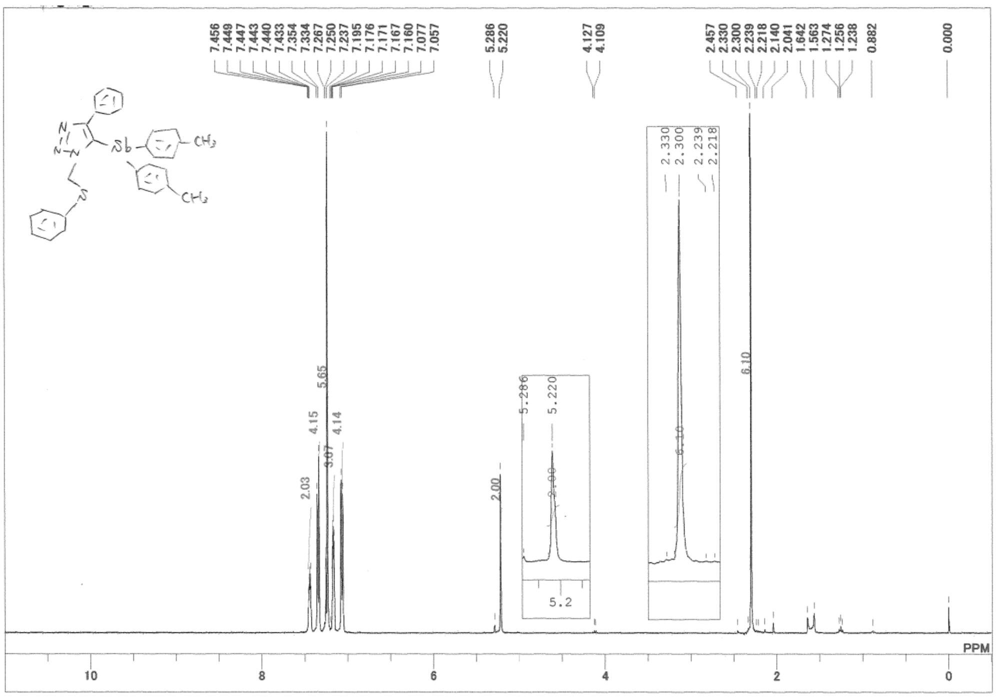

^{13}C NMR of **3g**

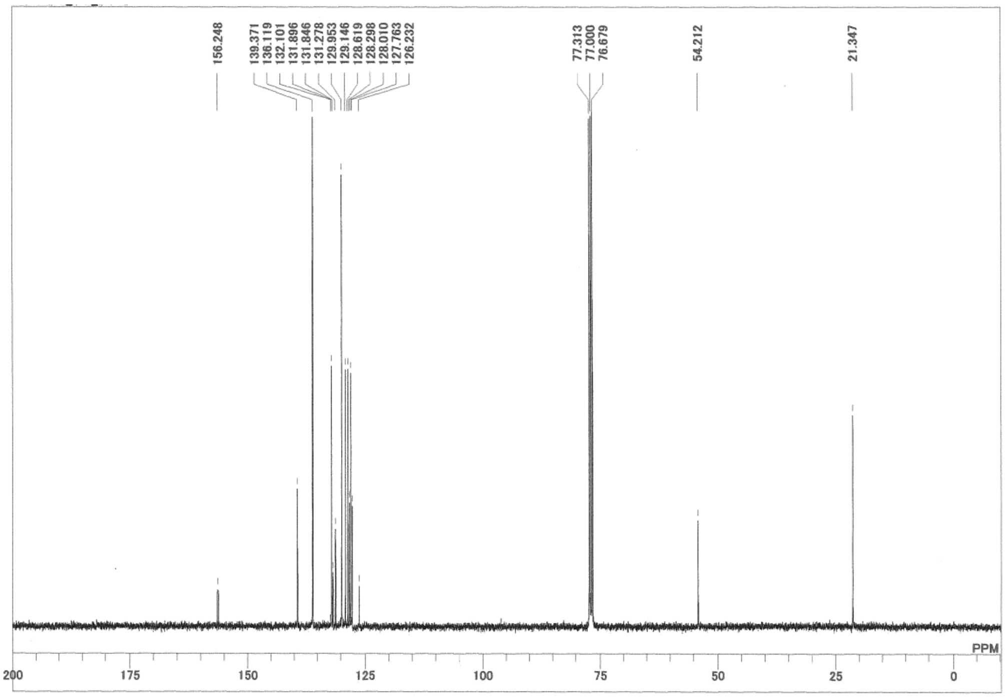

^{1}H NMR of **3h**

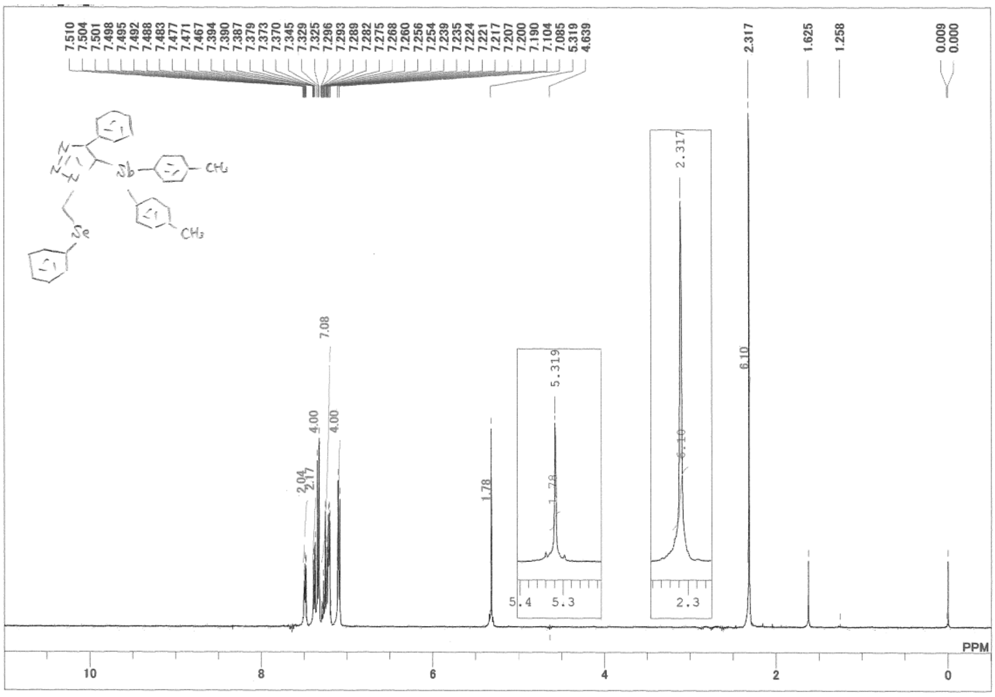

^{13}C NMR of **3h**

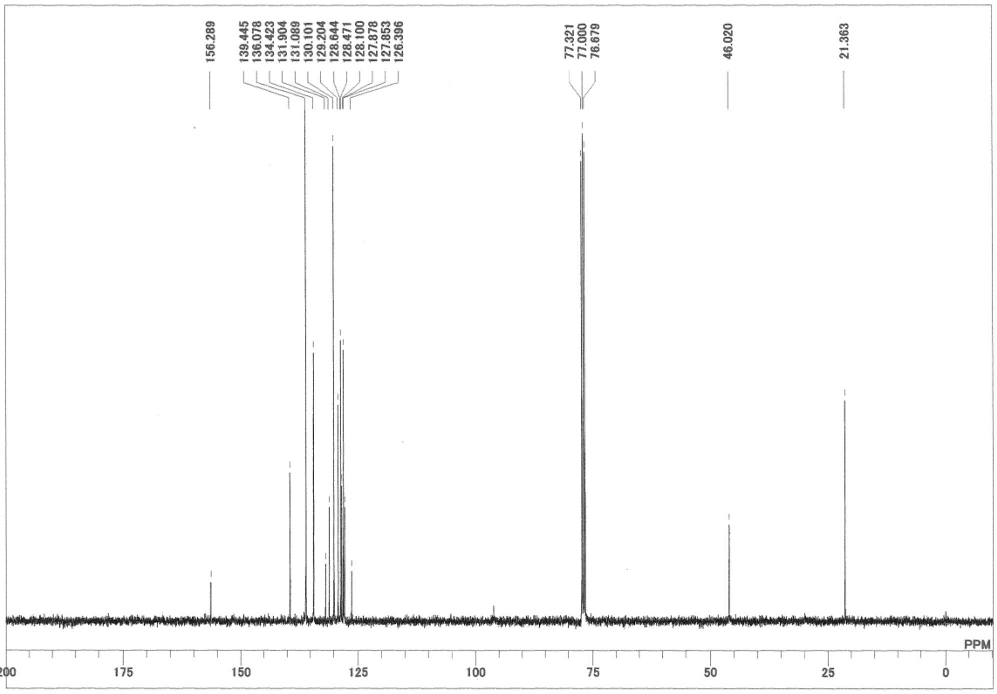

^{1}H NMR of **3i**

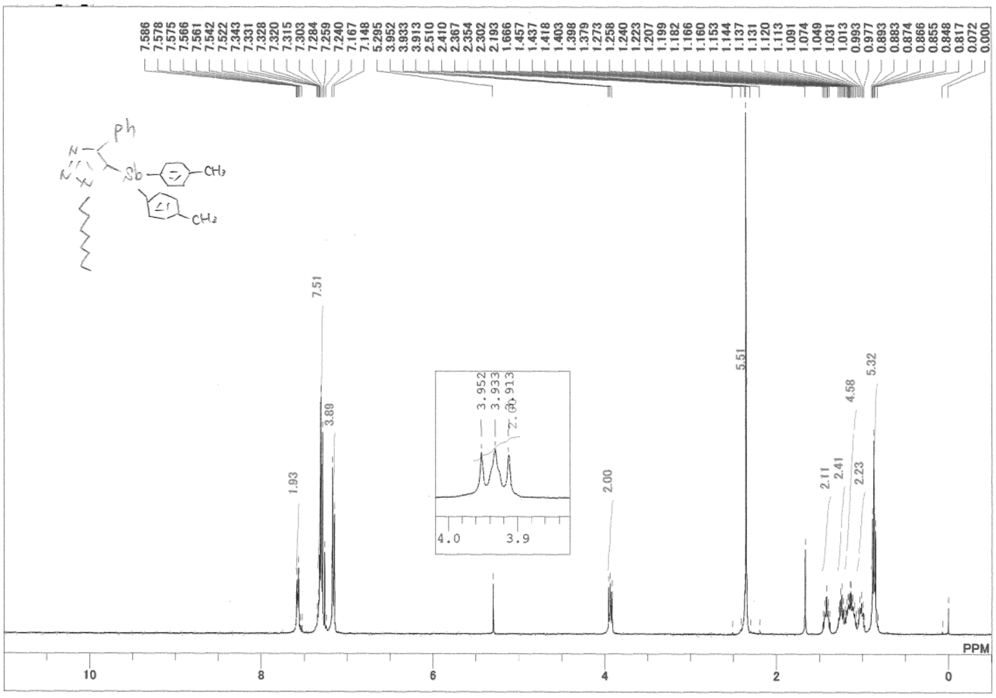

^{13}C NMR of **3i**

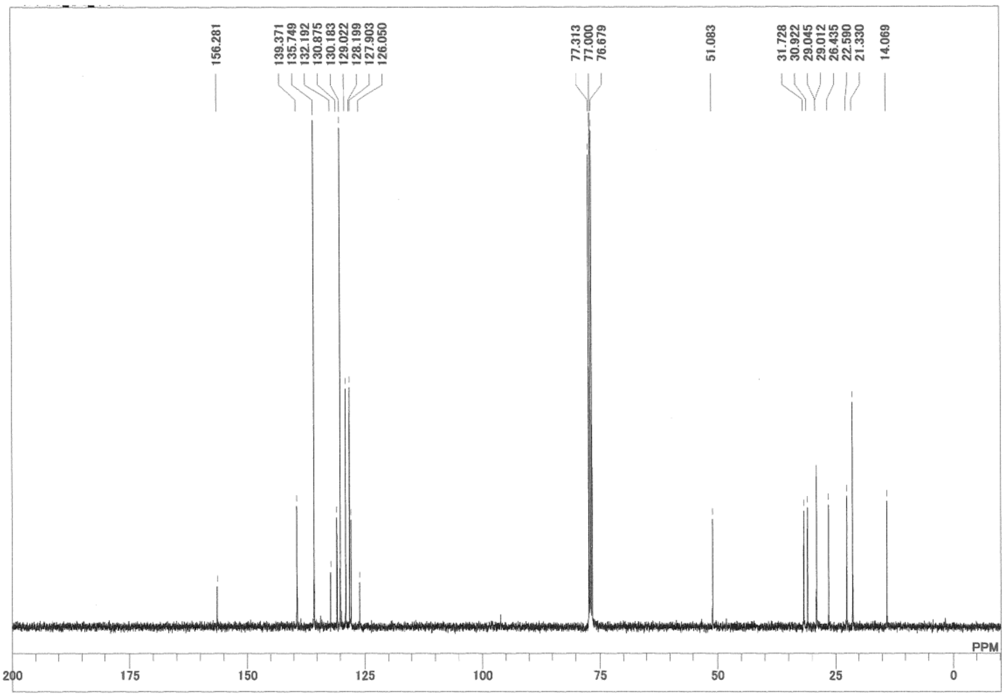

^{1}H NMR of **3j**

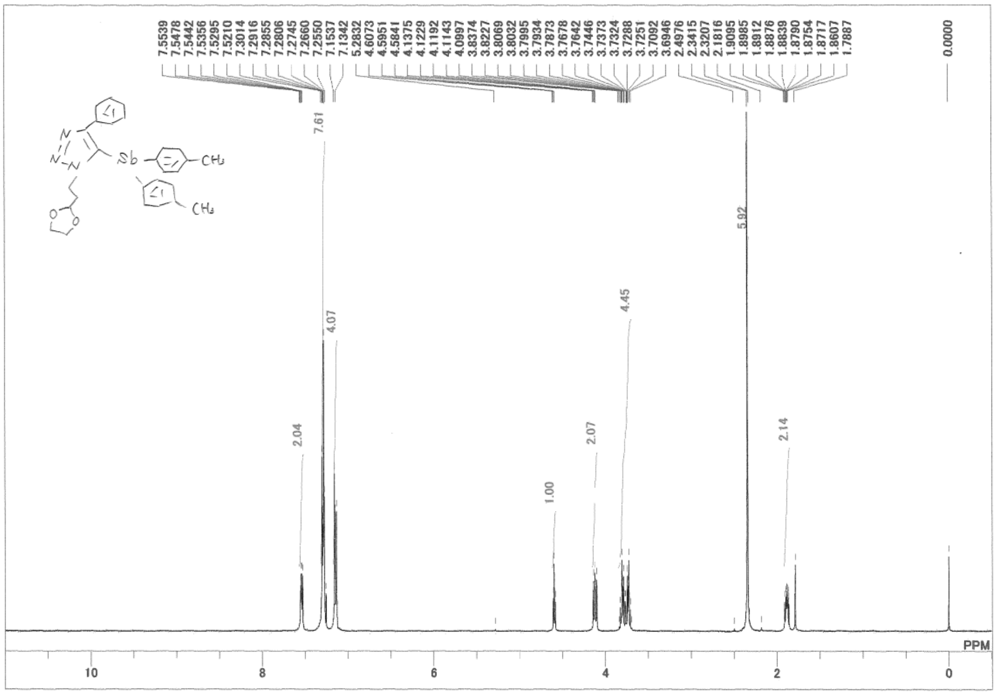

^{13}C NMR of **3j**

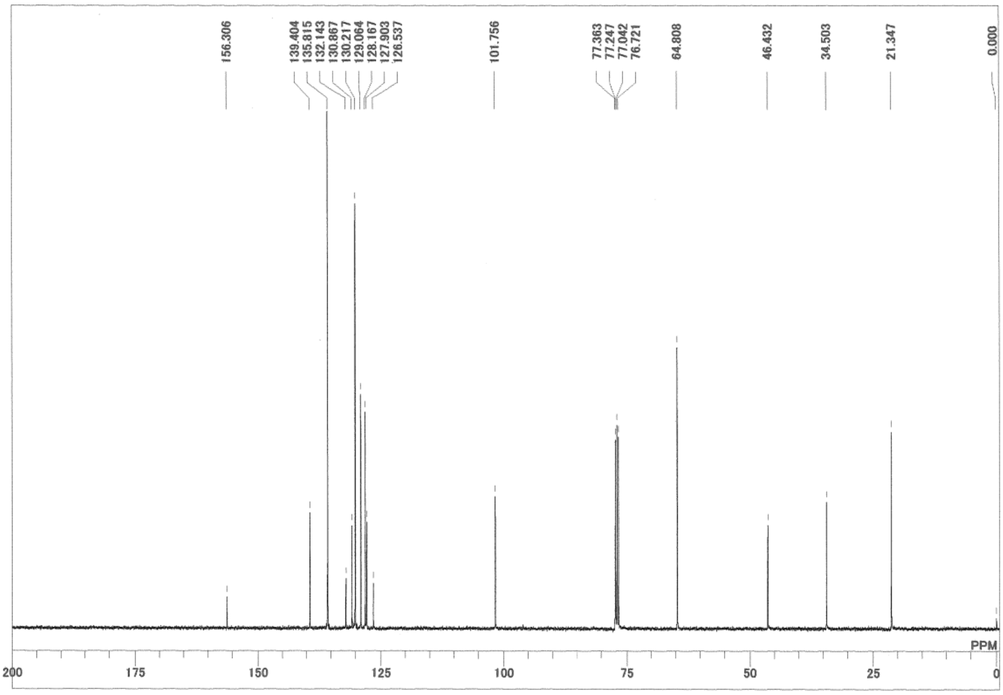

^{1}H NMR of **3k**

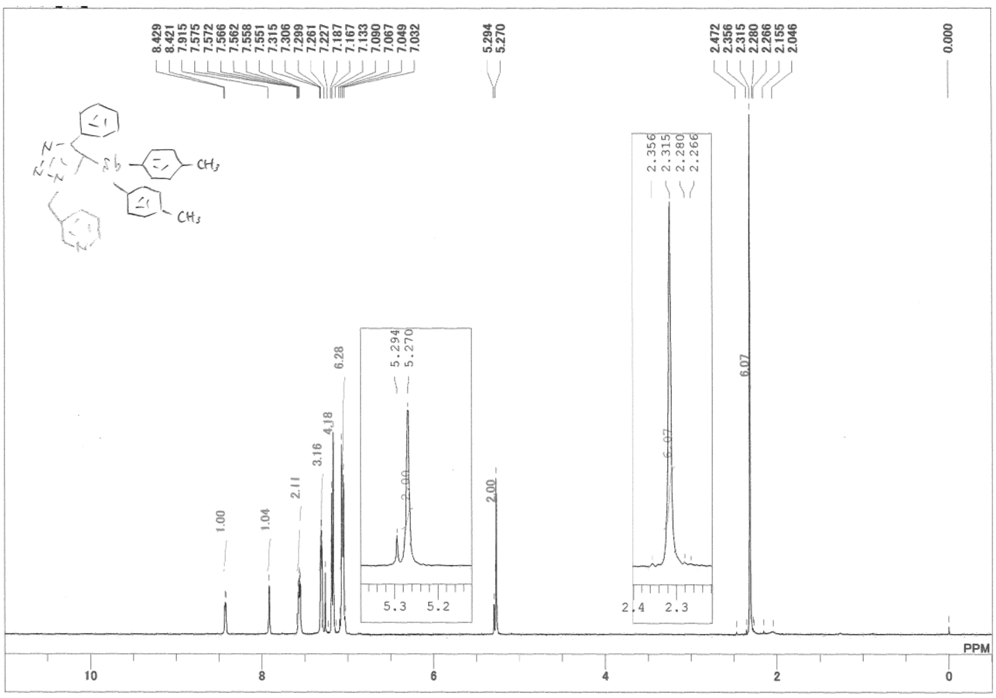

^{13}C NMR of **3k**

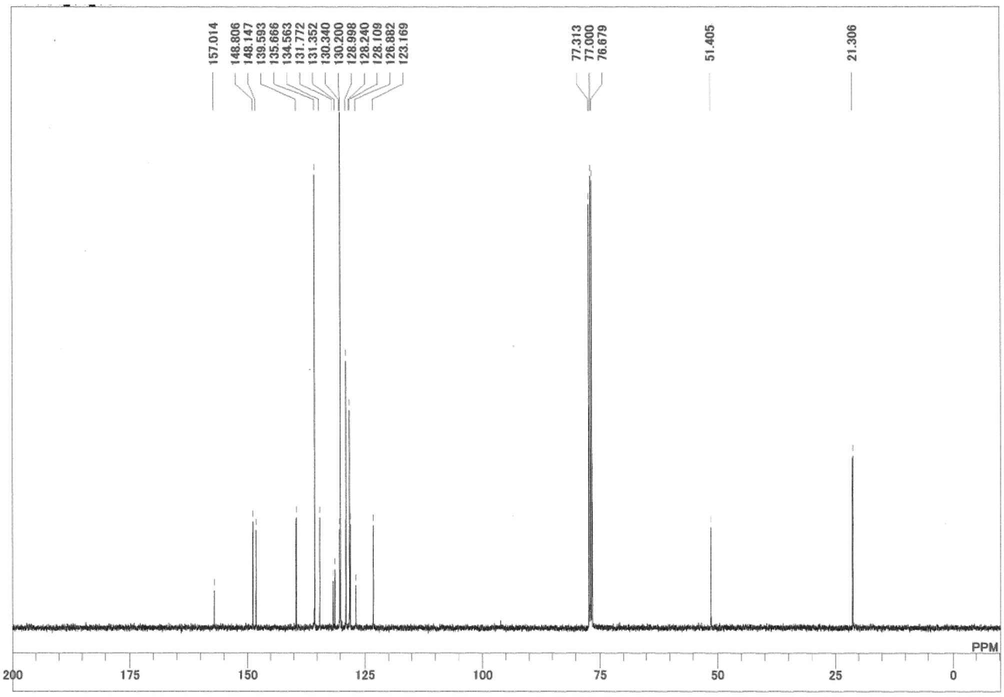

^{1}H NMR of **4**

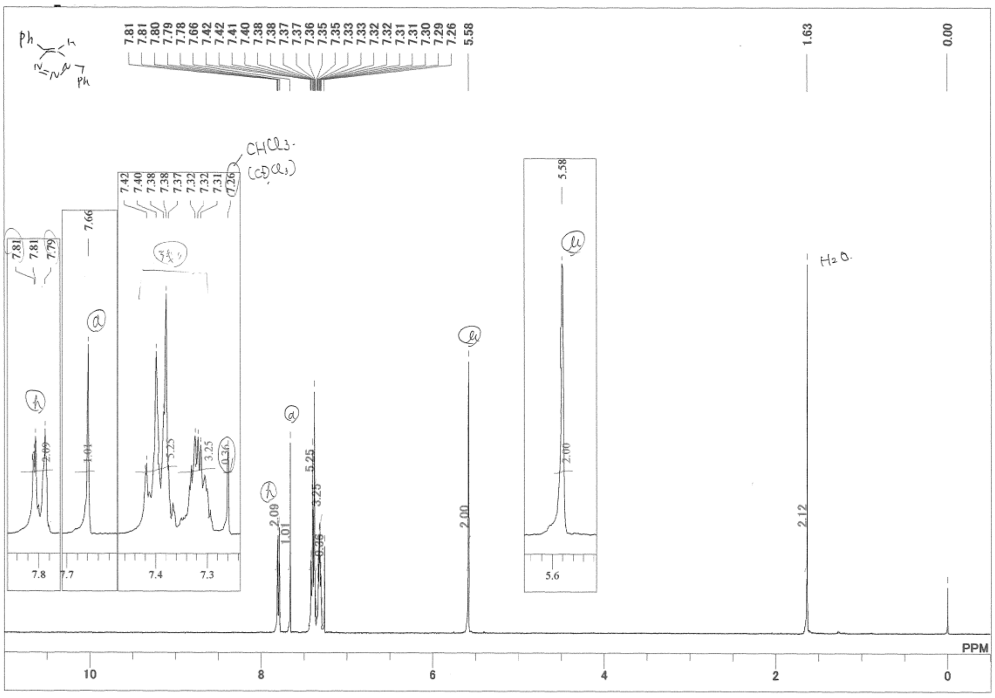

^{13}C NMR of **4**

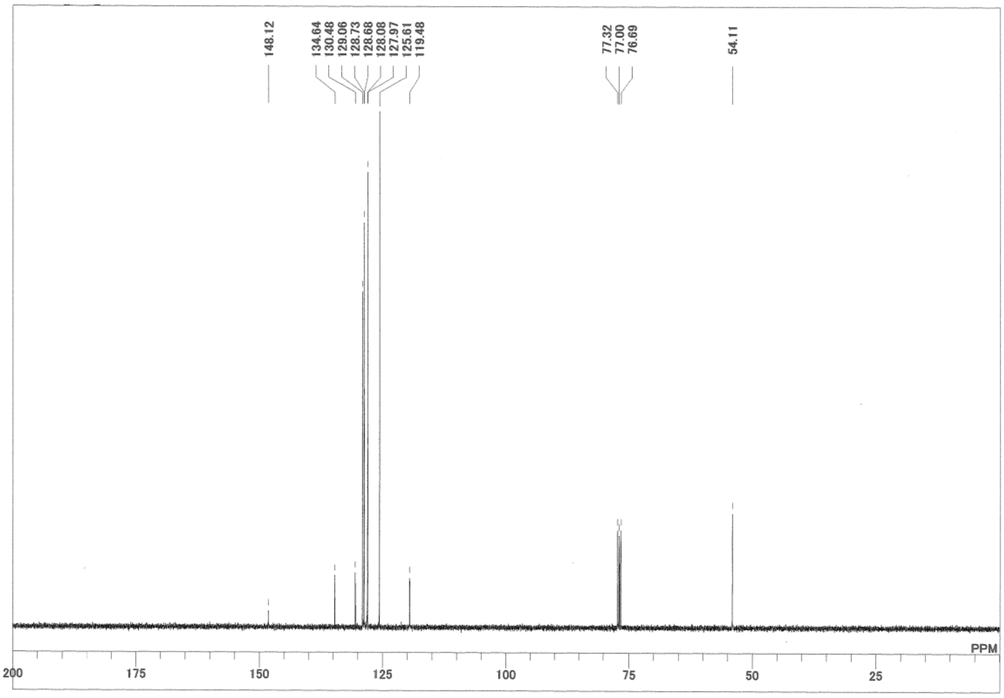

^{1}H NMR of **5**

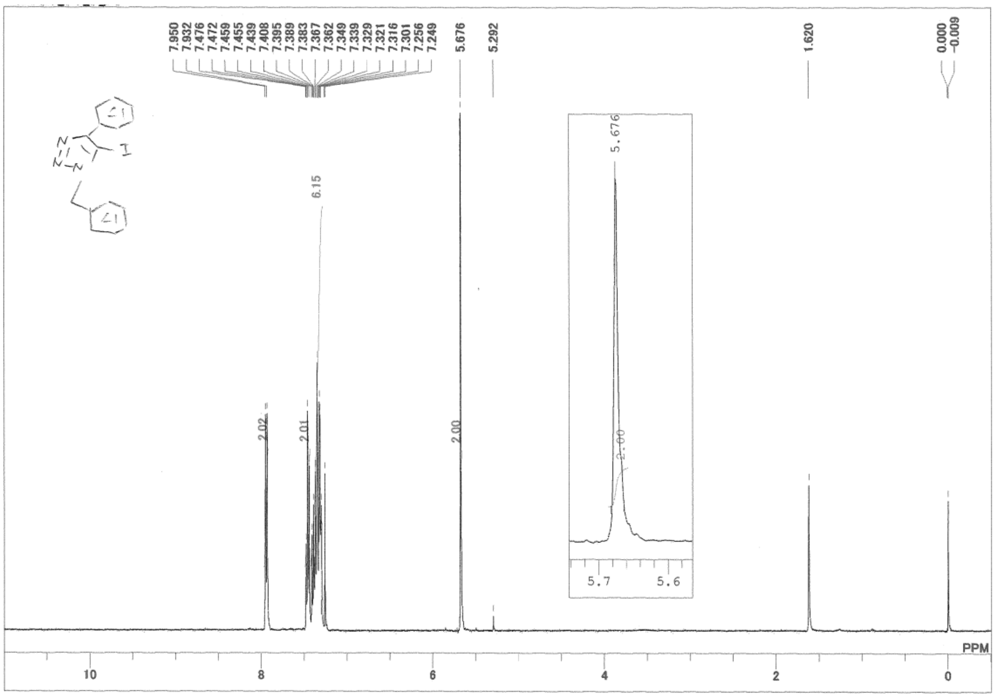

^{13}C NMR of **5**

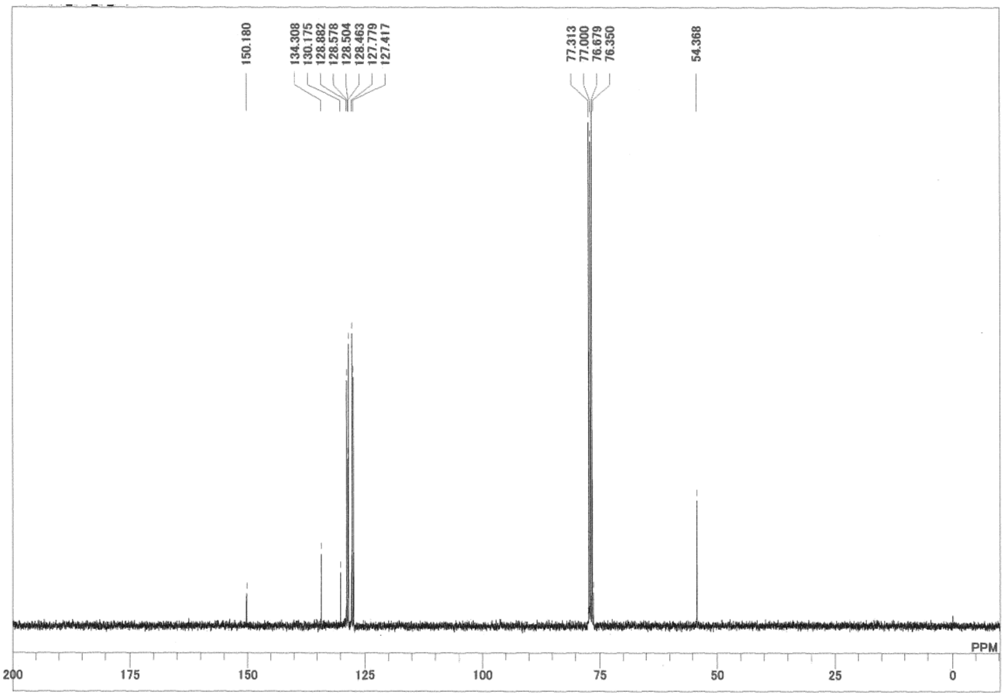

^{1}H NMR of **6**

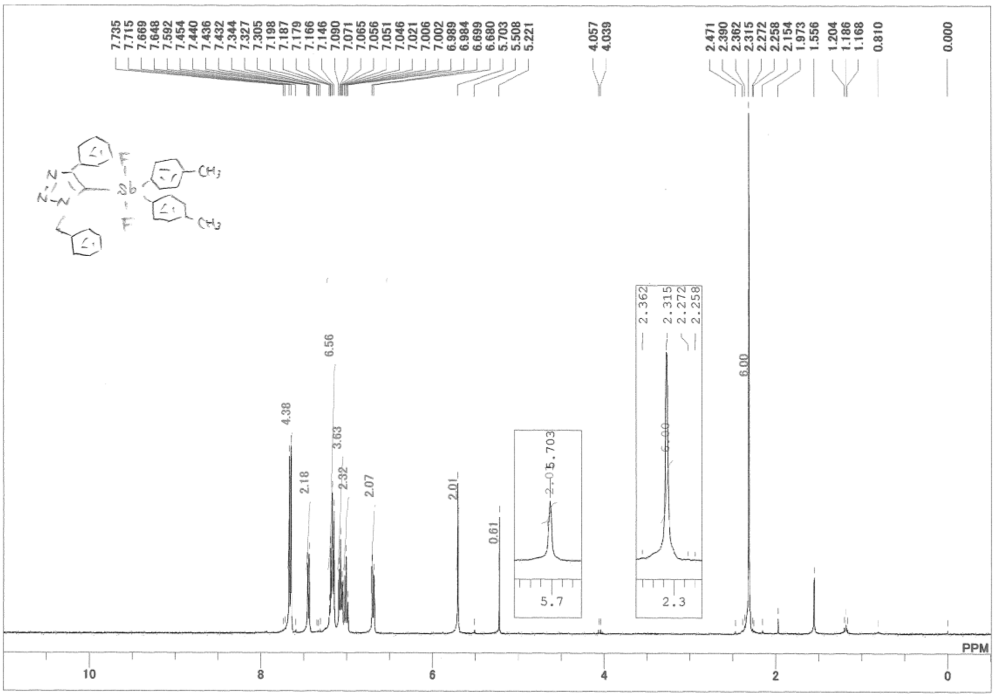

^{13}C NMR of **6**

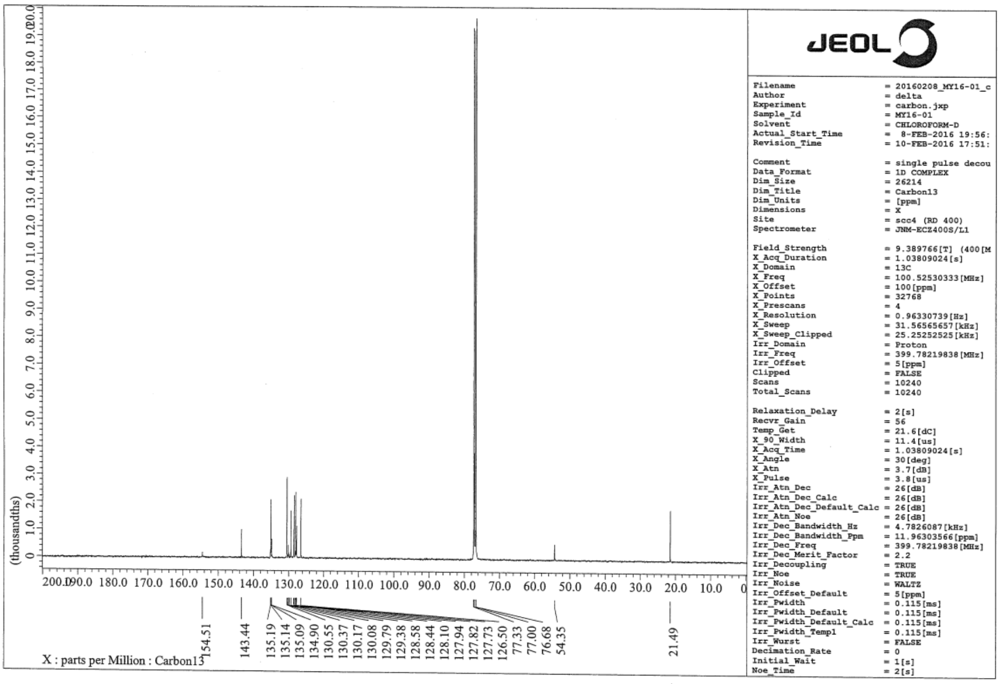

4

References

1. Hong, L.; Lin, W.; Zhang, F.; Liu, R.; Zhou, X. *Chem. Commun*. **2013**, *49*, 5589–5591.
2. Suzuki, T.; Ota, Y.; Ri, M.; Bando, M.; Gotoh, A.; Itoh, Y.; Tsumoto, H.; Tatum, P. R.; Mizukami, T.; Nakagawa, H.; Iida, S.; Ueda, R.; Shirahige, K.; Miyata, N. *J. Med. Chem*. **2012**, *55*, 9562–9575.
3. Alvarez, S. G.; Alvarez, M. T. *Synthesis* **1997**, 413-414.
4. Campbell-Verduyn, L. S.; Mirfeizi, L.; Dierckx, R. A.; Elsinga, P. H.; Feringa, B. L. *Chem. Commun*. **2009**, 2139–2141.
5. Huang, X.; Duan, D.-H. *Synlett* **1998**, 1191–1192.
6. Seus, N.; Saraiva, M. T.; Alberto, E. E.; Savegnago, L.; Alves, D. *Tetrahedron* **2012**, *68*, 10419–10425.
7. Brotherton, W. S.; Clark, R. J.; Zhu, L. *J.Org. Chem*. **2012**, *77*, 6443–6455.
8. Kakusawa, N.; Yamaguchi, K.; Kurita, J. *J. Organomet. Chem*. **2005**, *690*, 2956–2966.
9. Zhou, Y.; Lecourt, T.; Micouin, L. *Angew. Chem. Int. Ed*. **2010**, *49*, 2607–2610.
10. Sheldrick, G. M. *Acta Cryst*. **2008**, A64, 112–122.
11. Sheldrick, G. M. SHELXTL Version 2014/7. http://shelx.uni-ac.gwdg.de/SHELX/index.php.
